# Deep Learning Neural Networks Highly Predict Very Early Onset of Pluripotent Stem Cell Differentiation

**DOI:** 10.1016/j.stemcr.2019.02.004

**Published:** 2019-03-14

**Authors:** Ariel Waisman, Alejandro La Greca, Alan M. Möbbs, María Agustina Scarafía, Natalia L. Santín Velazque, Gabriel Neiman, Lucía N. Moro, Carlos Luzzani, Gustavo E. Sevlever, Alejandra S. Guberman, Santiago G. Miriuka

**Affiliations:** 1 LIAN-CONICET, FLENI, Ruta 9 Km 52.2 (B1625XAF), Belén de Escobar, Argentina; 2 Consejo Nacional de Investigaciones Científicas y Técnicas (CONICET), Buenos Aires, Argentina; 3 Laboratorio de Regulación Génica en Células Madre, Departamento de Química Biológica y Departamento de Fisiología, Biología Molecular y Celular, FCEN, Universidad de Buenos Aires, Argentina, Intendente Guiraldes 2160 (C1428EGA), Buenos Aires, Argentina; 4 IQUIBICEN, UBA/CONICET, Buenos Aires, Argentina

**Keywords:** deep learning, machine learning, artificial intelligence, computer vision, neural networks, pluripotent stem cells, embryonic stem cells, differentiation, light transmission microscopy, cell imaging

## Abstract

Deep learning is a significant step forward for developing autonomous tasks. One of its branches, computer vision, allows image recognition with high accuracy thanks to the use of convolutional neural networks (CNNs). Our goal was to train a CNN with transmitted light microscopy images to distinguish pluripotent stem cells from early differentiating cells. We induced differentiation of mouse embryonic stem cells to epiblast-like cells and took images at several time points from the initial stimulus. We found that the networks can be trained to recognize undifferentiated cells from differentiating cells with an accuracy higher than 99%. Successful prediction started just 20 min after the onset of differentiation. Furthermore, CNNs displayed great performance in several similar pluripotent stem cell (PSC) settings, including mesoderm differentiation in human induced PSCs. Accurate cellular morphology recognition in a simple microscopic set up may have a significant impact on how cell assays are performed in the near future.

## Highlights

• Deep learning (DL) neural networks can efficiently classify light microscopy images
• DL recognize PSC from 1 h differentiating epiblast-like cells
• DL discriminates images of mouse ESCs in 2i + LIF from those grown in FBS + LIF
• DL accurately classifies images of human iPSCs from those of early mesodermal cells

## Graphical Abstract

**Figure:**
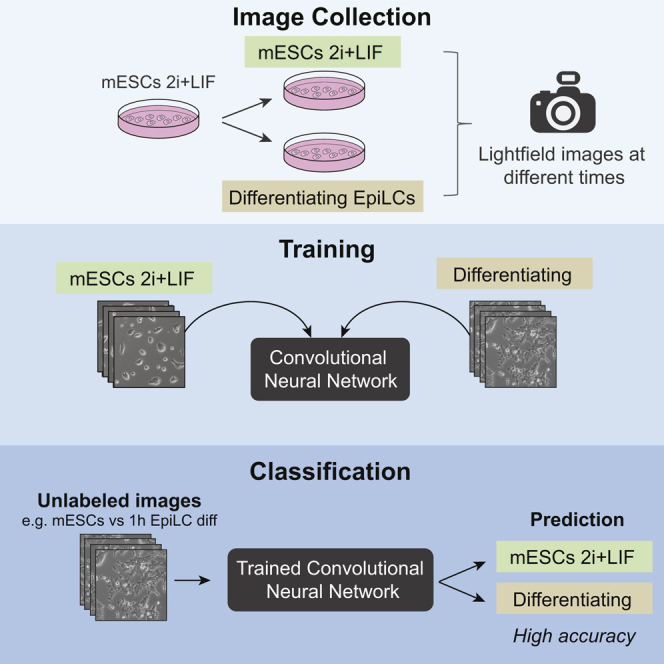

## Teaser

In this article, Miriuka and colleagues show that deep learning convolutional neural networks can be trained to accurately classify light microscopy images of pluripotent stem cells from those of early differentiating cells, only minutes after the differentiation stimulus. These algorithms thus provide novel tools to quantitatively characterize subtle changes in cell morphology.

## Introduction

Major advances in artificial intelligence have occurred in recent years. New hardware with significantly increased calculus capacity and new software for easier application of complex algorithms allow now to apply powerful predictions in many fields. Neural networks have particularly benefited from this progress. With proper design, these algorithms are highly efficient for machine learning classification tasks. The term *deep learning* (DL) has been coined for these neural networks with extremely high amount of calculations (LeCun et al., 2015). DL has proved to be particularly useful in computer vision, where it allows image recognition by learning visual patterns through the use of the so-called convolutional neural networks (CNNs) (Camacho et al., 2018, Cao et al., 2018, Voulodimos et al., 2018). Roughly, a CNN processes all numbers composing a digital image and identifies the relationship between them. These relations are different according to the different objects found in the image, and in particular at the edges of these objects. The process of finding the optimal weights that makes these predictions is a key step in CNN training. This task is performed through the application of very large amounts of weighted regressions, which can take very high computational requirements, a long time, and a significant number of images. However, once trained, applying the neural network training to get predictions is relatively fast and allows almost instant image recognition and classification. For example, powerful CNN training now allows tasks as diverse as autonomous car driving and face recognition in live images.

The expansion of CNNs to biomedicine and cell biology is foreseen in the near future (Camacho et al., 2018). Several recent reports highlight the possible application of DL in cell and molecular biology (Ching et al., 2018). Fluorescent staining prediction (Christiansen et al., 2018), bacterial resistance (Yu et al., 2018), or super-resolution microscopy improvement (Ouyang et al., 2018) are some of the successful applications that have been described. Based on what has been developed so far using deep learning, the experimental assays where visual pattern recognition is necessary may soon be substantially transformed.

One of the areas that could benefit from the advances in DL is the field of mammalian pluripotent stem cells (PSCs). These cells have the remarkable capability to differentiate to all the cell types of the organism, which has made them gain a lot of attention in areas such as regenerative medicine, disease modeling, drug testing and embryonic development research. There are two main types of PSCs: (1) embryonic stem cells (ESCs), which are derived from the inner cell mass of peri-implantation blastocysts, and (2) induced PSCs (iPSCs), which are similar to ESCs, but originate through cell reprogramming of adult terminally differentiated cells by overexpressing core pluripotency transcription factors. PSC differentiation is a highly dynamic process in which epigenetic, transcriptional, and metabolic changes eventually lead to new cell identities. These changes occur within hours to days, and even months, and are generally identified by measuring gene expression changes and protein markers. These assays are time consuming and expensive, and normally require cell fixation or lysis, thus limiting their uses as quality-control evaluations necessary for direct application of these cells to the clinic. In addition to these molecular changes, PSC differentiation is followed by an important morphological transformation, in which the highly compact PSCs colonies give rise to more loosely organized cell structures. Although these morphological changes can be quite evident to the trained human eye, they are inherently subjective and thus are not used as a standard and quantitative measurement of cell differentiation.

In this paper we test the hypothesis that CNNs are able to accurately predict the early onset of PSC differentiation in plain images obtained from transmitted light microscopy. For this purpose, we used a model in which mouse ESCs (mESCs) maintained in the ground state of pluripotency were differentiated to epiblast-like cells (EpiLCs), which are in the formative state of pluripotency (Hayashi et al., 2011, Smith, 2017). This experimental system, which recapitulates early events that occur during embryonic development, is very efficient and it is completed in only 24–48 h. By applying CNN training at different times from the onset of differentiation, we show that the trained CNN can identify differentiating cells only minutes after the differentiation stimuli. We show that CNNs can also be trained to distinguish mESCs in the ground state of pluripotency from mESCs maintained in serum and leukemia inhibitory factor (LIF) (fetal bovine serum [FBS] + LIF), a culture condition routinely used to maintain mESCs in the naive undifferentiated state but that displays higher cell heterogeneity and increased expression of differentiation markers. Furthermore, CNNs were also able to accurately classify undifferentiated human iPSCs (hiPSCs) from early differentiating mesodermal cells. We believe that accurate cellular morphology recognition in a simple microscopic set up may have a significant impact on how cell assays are performed in the near future.

## Results

### Initial Training

**Figure 1:**
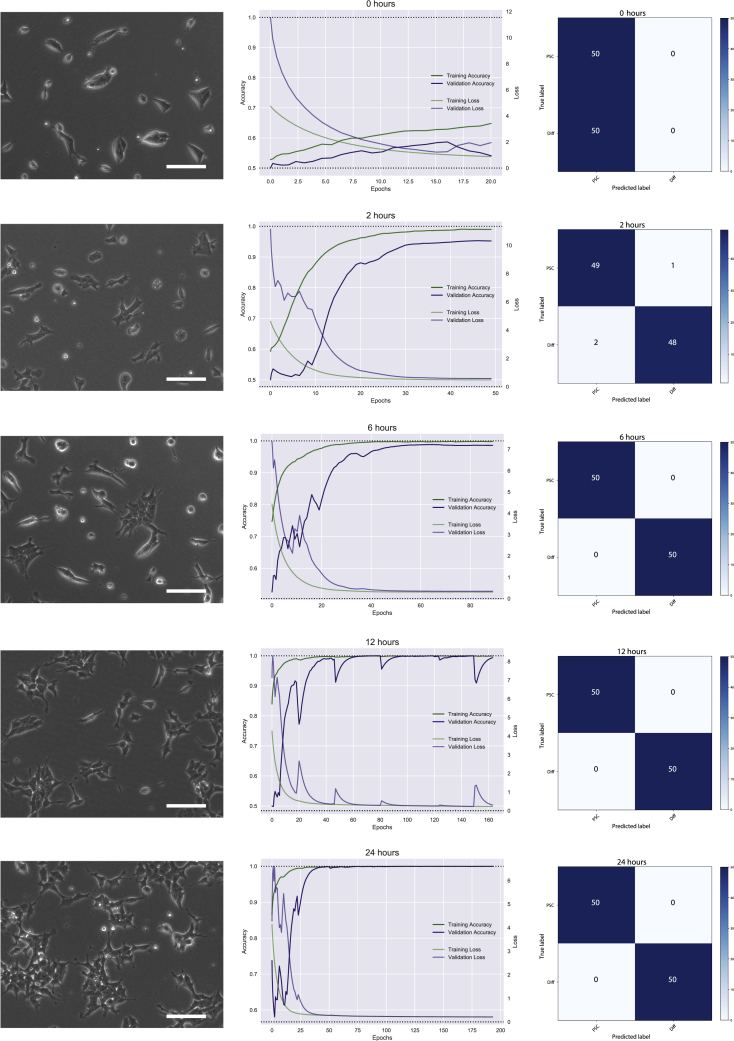
Convolutional Neural Network Training Images were taken from pluripotent and differentiating 46C mESCs and fed into a ResNet50. On the left panels it can be seen how colonies acquire a spindle shape as time progresses. Scale bars, 100 μm. Early changes are present at 2 h after the onset of differentiation. In the center column, each panel presents the training and validation accuracy, as well as the training and validation loss. As time from the onset of differentiation shortens, the training becomes less confident. Although an acceptable training is reached at 2 h, validation accuracy does not reach a value close to 1. No training is obtained at time zero. On the right columns, confusion matrix graph shows the results of testing the CNN on 100 independent images. Accuracy is at the top at 6, 12, and 24 h, falling to 0.97 at 2 h.

Early after the onset of differentiation, mESCs rapidly changed their morphology. By 24 h, they acquired a substantial volume of cytoplasm, some cells detached from each other, and colonies spread with a spindle shape form (Figure 1). We initially took images at 0, 2, 6, 12, and 24 h and trained a CNN based on the ResNet50 architecture (He et al., 2015), a well-known CNN architecture with proved efficacy. Approximately 800 images per group were provided to the network for each condition, plus 200 per group for testing during training, and 50 images per group for final, independent validation. Approximately 800 images were provided to the network for each condition. As expected, at 0 h (images taken immediately before differentiation onset) the training accuracy was compatible with a random state of prediction between the two states, although some training is seen as the epoch cycles learns from itself. Thereafter, the trained network was able to predict with high level of accuracy in both training and validation samples. Independent test accuracy was 1 at 6, 12, and 24 h, and 0.97 at 2 h after the onset of differentiation.

**Figure 2:**
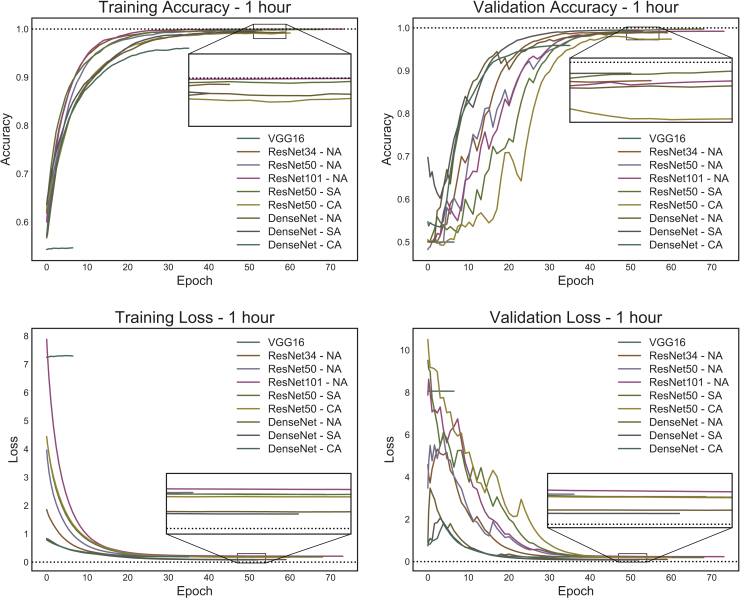
Training and Validation Accuracy and Training and Validation Loss At 1 h of Differentiation Several CNNs were used to train images at 1 h. All networks achieved results close to 100% of accuracy. Insets in all panels shows details of the stable phase. DenseNet architectures showed validation accuracies with a steeper curve, although reaching similar values than ResNet, in particular when simple image augmentation was used. Validation loss was slightly lower when training was done with DenseNet architectures. VGG16, a shallower architecture, could not be trained. All models were run with the same initial weights. NA, no image augmentation; SA, simple image augmentation; CA, complex image augmentation.

After getting this encouraging level of accuracy, we collected more measurements to improve the network's performance and took a new set of images at 1 and 2 h of differentiation. Also, we had previously observed that, with the initial cell density (30 × 10 cells/cm), there were many image slices with very few cell colonies, and hence we hypothesized the CNN may not extract enough features for proper training, in particular at early time points. Therefore, we increased the initial cell seeding number up to 60 × 10 cells/cm. We also increased the number of images feeding to the CNN to approximately 1,000 per group (250 images before slicing). We also assessed variants in the architecture of the CNN. We tried different numbers of hidden layers in ResNet (34 and 101), and with another deep neural network with different architecture (DenseNet [Huang et al., 2016]; for a comprehensive review about networks consult [Zahangir Alom et al., 2018]). Finally, we compared different approaches to preprocess images in order to increase the model performance, a process known as *image augmentation*. Figure 2 shows the results at 1 h after the onset of differentiation. We achieved a very high training accuracy with all trained networks (Table S1). We noticed that increasing image preprocessing did not necessarily increased accuracy. Also, increasing the depth of the network or its complexity may not improve results. In our stem cell model, best performance was achieved with ResNet50 with none or simple image augmentation (ResNet-SA) and with DenseNet with simple augmentation (DenseNet-SA). Of note, DenseNet with simple and without augmentation got the lowest validation loss, a measure of training performance. We then used the successful networks ResNet50-SA and DenseNet-SA to train images taken at 2 h from the onset of differentiation, and accuracy was again at 100% (Figure S1). Importantly, we found similar results when using a different mESC cell line, the E14-derived Ainv15 cells. At 1 h, training reached approximately 85% accuracy, peaking to more than 99% after 8 h of differentiation to EpiLCs (Figure S2). The slight difference in accuracy at 1 h with respect to the 46C mESCs might be due to the fact that Ainv15 cells grow more loosely attached to the plate, forming tridimensional colonies that thus take longer to change in morphology even when visually inspected. These results further validate the applicability of CNN classification on early-differentiating mESCs, and also highlight that variability between different cell lines can be efficiently quantified.

All tested CNNs are very deep in terms of number of layers, with a significant number of hidden layers and a huge calculation burden. To test if other architecture with less layers was able to train to a similar extent, we ran the same analysis with VGG16 (Simonyan and Zisserman, 2014), a shallower network, adding simple image augmentation. However, the training of this neural network was unsuccessful with our image set as it reached the futile training function (Figure 2). Eventually, if left running, VGG16 may train the images, but it would take much longer time and resources.

### Optimizers

Learning rate (LR) is critical for training neural networks. LR adjusts, at every training cycle (called *epoch*), the rate at which the network weights will be modified in order to find the minimum and best loss. Several algorithms (usually known as *optimizers*) that adjust LR decaying according to training have been developed. In all previous analyses we used the Adam algorithm, but several others have been proposed (Ruder, 2016). We compared them in a limited training of 40 epochs using ResNet50. We found that Adam, Adamax, and Adagrad were equally good, as opposed to Nadam and RMSprop, which both trained at a lower speed (Figure S3A). We were unable to train the neural network using stochastic gradient descent, although we cannot rule out that, with proper adjustment, this algorithm would eventually train the image set. Figure S3B shows how LR adjusts itself as epochs progress to the end of training. By the end of training, LR is a small fraction of the initial one, and then allows finding of the minimal loss. Several options can then be used to find the proper LR to train the model.

### Minimal Number of Images Required

DL neural networks require a significant amount of information to identity features, and thus many images are usually needed. How many images are indeed needed for optimal training is usually unknown and hard to predict, and may significantly change between experiments depending on the sort of images. For our 1-h training of mESCs, we used 2,120 images (920 images in the 2i + LIF group and 1,120 in the differentiation group). We then successively trained ResNet50-SA with less images (100 less per group in each retrain) to identify a minimal number needed to train. The results show that, as the number of images decreased, all parameters of training efficiency also decreased (Figure S4). Underfitting, represented by a much lower validation accuracy than training accuracy, was observed when 1,400 images or less are used. A progressive improvement is seen as images are increased up to the full number available. Validation accuracy and loss reach the highest level with the full set of images. Of note, independent test on these analyses showed accuracy values over 0.9 in all trainings, except those with very low image numbers (200 and 400, data not shown). These analyses suggest that careful decision should be made when choosing the number of images needed, as a lower number can produce acceptable results, but still underfitted and not according the training possibilities.

### CNN Image Representation

**Figure 3:**
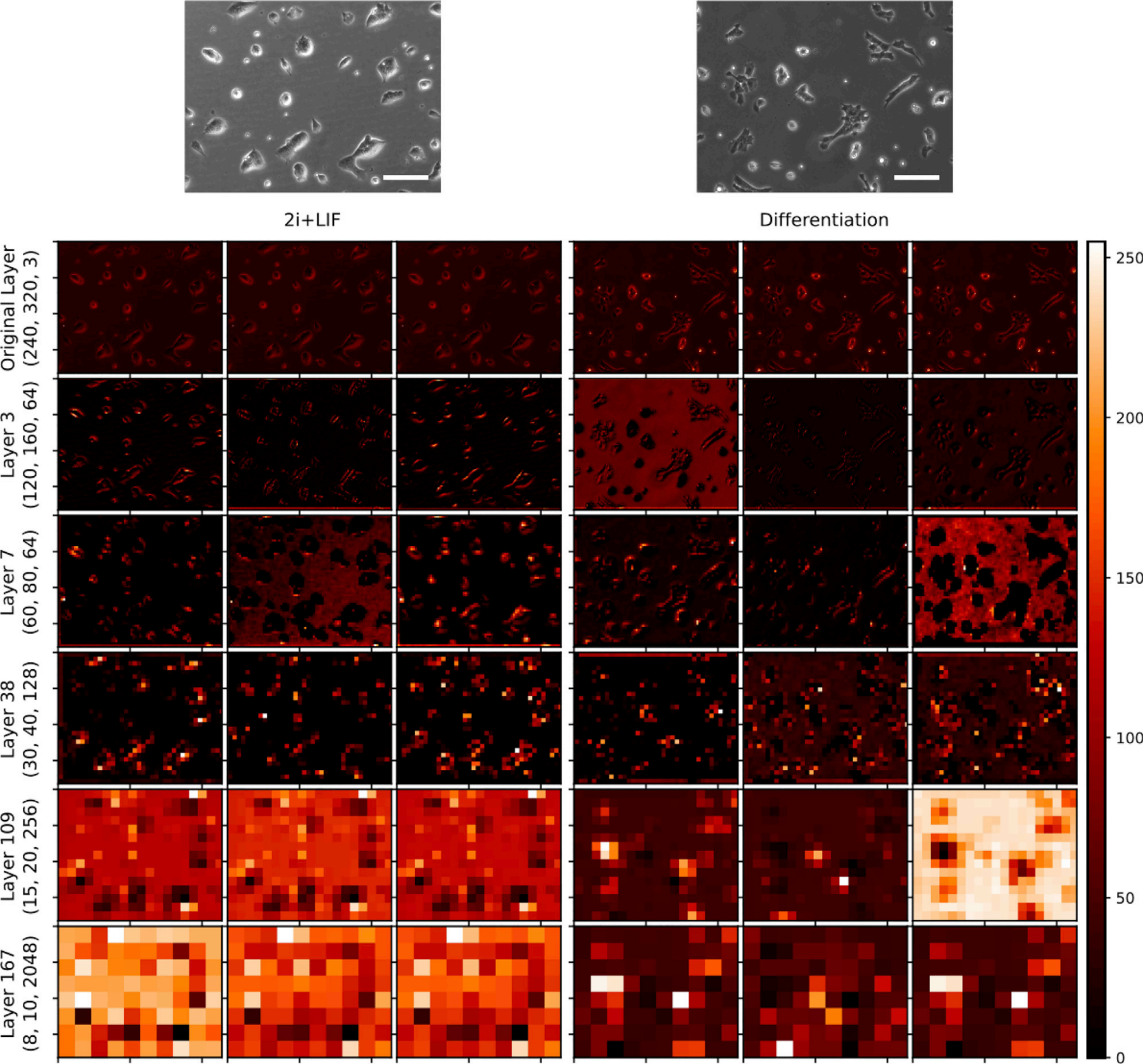
CNN Image Representation The process of CNN learning involves reducing the size of the image and at the same time increasing depth. From a dimension in the original layer (display on top) of 480 rows, 640 columns, and 3 layers (480, 640, and 3), the networks progressively go down to a final layer of 8 × 10 × 2,048. The figure represents this process for one image of each group (2i + LIF and 1 h after onset of differentiation). Only three figures of some of the activations layers are shown. Color bar scales the image activations from 0 to 255. The learning process ends up by providing the last 2,048 figure weights of each image to a loglinear regression, which evaluates the two possible outcomes (2i + LIF versus differentiation), and hence a probability is given. Convolutions are known to particularly recognize borders of objects, and this is observed in the intermediate layers, where cell colonies are defined by their shape. These borders are then translated into specific patterns in the final layer. In the last row it can be observed patterns of activations that are different in 2i + LIF and differentiated 2i + LIF. The repetition of these activation patterns allows image classification. Scale bars, 100 μm.

Training a neural network involves a huge amount of calculations in a series of so-called “hidden” layers. The intermediate calculation values in the hidden layers can be obtained and used to build up intermediate images. It is possible then to plot what the CNN is actually doing by translating the activation layers into pixels, and, hence, to get an insight on how the CNN does *see* an image and how it performs its classification task. ResNet50-SA has a total of 168 layers, with 49 of them containing *activations* (that is, representation of the pixels) (Data S1). Figure 3 shows the representation of the activations in some of the hidden layers of images trained with ResNet50-SA. At the top of the panel both original images from 2i + LIF and differentiating cells are seen. The dimensions on these original images are as given to the CNN: 480 rows by 640 columns by 3 layers (480, 640, 3). The last dimension corresponds to the red, green, and blue (RGB) channels. Of note, we fed the CNN with images in greyscale, although with the three-color layers. When CNNs were trained in greyscale (240 × 320 × 1), no differences in accuracy or loss were seen. This original size is immediately reduced to 240 × 320 at the entry of the neural network. As the CNN deepens, the activation layers are progressively smaller in the first two dimensions and bigger in the last one. By the end of the network, the final activation layer has a small size (8 × 10) but high depth (2,048 channels). This last activation’s layer's weights are fed to a binary sigmoid function for prediction. Hence, 80 pixels in 2,048 channels by 256 possible values gives almost 42 × 10 possible pixel variations for each image. The repetitive relation of these values in all images fed to the CNN provides the patterns used for image identification.

DenseNet has a different architecture, with 140 total layers and 39 activation layers (Data S2). An example of some activation layers in DenseNet are shown in Figure S5. In this network, the final layers are bigger with lower depth (dimensions 60, 80, 12). Depth expands and then contracts in DenseNet, as opposed to ResNet50. Representation of 2i + LIF cells and differentiating cells show that activations change with different cell morphologies; in 2i + LIF they are rounder than differentiating cells.

### Biological Changes during the First Hours of Differentiation

Given the high accuracy of predictions made in such a short time after the onset of differentiation, we next decided to evaluate what biological changes could be detected in these early time points.

**Figure 4:**
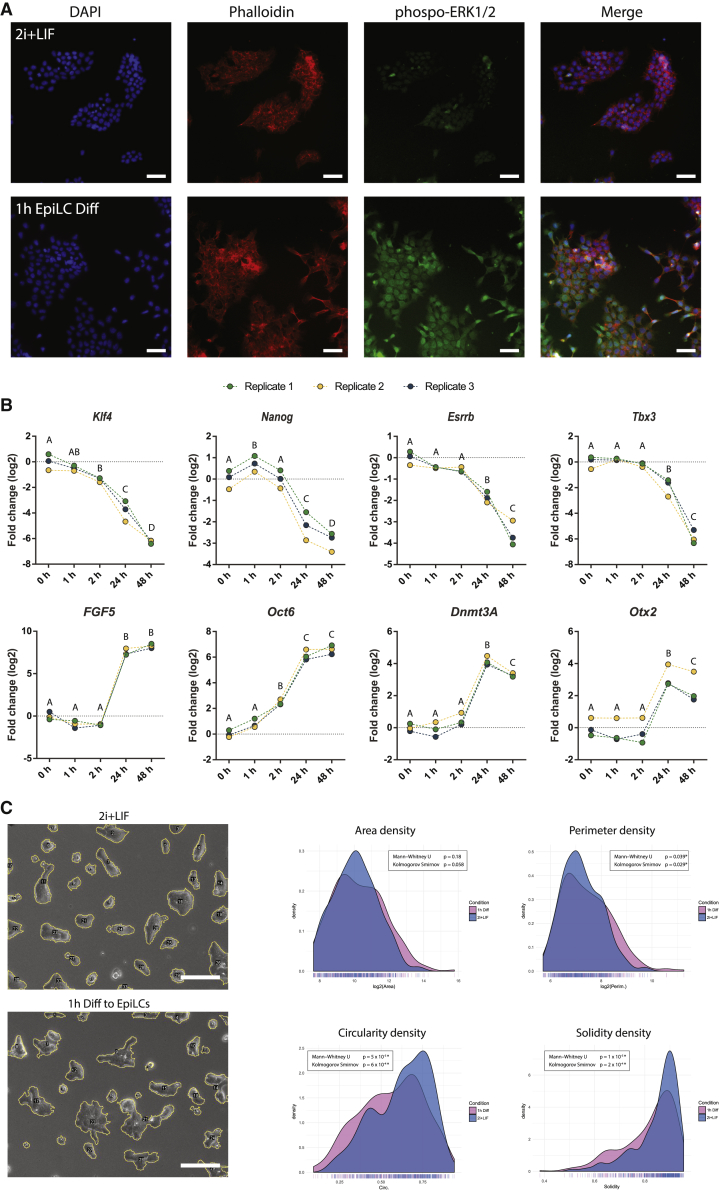
Gene Expression, Cell Signaling, and Morphological Differences of Early Differentiating EpiLCs (A) Representative immunostainings comparing mESCs cultured in 2i + LIF and after 1 h of induction to EpiLCs (1 h EpiLC Diff). Cells were evaluated for ERK1/2 phosphorylation and for the re-organization of the actin cytoskeleton (phalloidin). Nuclei were stained with DAPI. Scale bars, 50 um. (B) qRT-PCR analysis of primed and naive pluripotency markers in 2i + LIF or after 1, 2, 24, and 48 h of EpiLC induction. Results are presented displaying the log2 transformed values of the three independent biological replicates relative to time 0 h (2i + LIF), to clearly show the high reproducibility of the results. Letters indicate significant differences between groups (p < 0.05) by randomized block design ANOVA. (C) Morphological analysis of cell colonies grown in 2i + LIF or after 1 h induction to EpiLCs. Colonies were automatically detected from 20 wide-field images (see Experimental Procedures) and morphological properties were recorded and analyzed. Left images display representative colony segmentations for 2i + LIF and 1 h EpiLCs Diff. Scale bars, 100 μm. Right charts display the distribution density for the indicated morphological variables comparing 2i + LIF and 1 h EpiLC Diff (n = 326 and 291 colonies, respectively). Statistical differences were evaluated using the Kolmogorov-Smirnov and the Mann-Whitney U test. ^∗^p values < 0.05 were considered statistically significant.

The activation of the MEK/ERK signaling pathway is one of the key events that leads to mESC differentiation (Nichols et al., 2009). Thus, we analyzed whether this pathway was already activated after 1 h of differentiation by assessing the levels of phospho-ERK. Interestingly, most of the early-differentiating cells already displayed an increased level of nuclear and cytoplasmatic phospho-ERK. On the contrary, for mESCs in 2i + LIF, only cells in the M phase of the cell cycle showed a phospho-ERK signal, as reported previously (Shapiro et al., 1998) (Figures 4A and S6). These results corroborate that differentiation signals are rapidly transduced into cells.

We next wondered whether the activation of the differentiation signals led to the modification of the transcriptional profile of the cells at this short time. We assessed the expression of several naive and primed pluripotency markers at 1, 2, 24, and 48 h of differentiation. As expected, the naive pluripotency markers *Klf4*, *Nanog*, *Esrrb*, and *Tbx3* were significantly downregulated at 24 and 48 h, while the primed markers *FGF5*, *Oct6*, *Dnmt3A*, and *Otx2* were upregulated (Figure 4B). Interestingly, we found that during the first 2 h of differentiation there were minor but significant changes in the expression of the naive markers *Klf4* and *Nanog*, as well as in the differentiation marker *Oct6*. Consistent with our results, KLF4 has recently been shown to be phosphorylated by phospho-ERK, which induces its exit from the nucleus affecting its own transcription very early in the differentiation process (Dhaliwal et al., 2018). The behavior of *Oct6* is also supported by our previous work, where we showed that *Oct6* is rapidly induced during exit from ground state pluripotency in another mESCs cell line (Waisman et al., 2017). The slight but consistent transitory upregulation of *Nanog* during the first hour is intriguing, and we believe this might be a consequence of a re-organization of regulatory elements in its promoter region, although further research needs to be done. Overall, these results indicate that within this short frame of time mESCs begin to modify their transcriptional profile.

It is thus evident that there are several molecular signatures already present at 1 h from the onset of the differentiation stimuli. However, due to the nature of the images used to train the CNN, the morphological transformation of cell colonies is the only parameter that the neural network can detect and use as input for making predictions. As we have previously mentioned, it has been described that CNNs specifically recognize shape borders of the object in the images (Krizhevsky et al., 2017). To further study these changes at the molecular level, we analyzed the organization of the actin cytoskeleton by staining cells with phalloidin. Interestingly, fluorescent images clearly show that differentiating cells rapidly re-organize the distribution of the actin filaments, with many cells displaying minor spindles protruding from their surface (Figure 4A).

To get more insight into the morphological differences between the two conditions under study, we finally analyzed the morphological properties of hundreds of colonies in the undifferentiated state or subjected to 1 h of differentiation. We focused on parameters such as colony area, perimeter, circularity, and solidity, the latter being a measurement of how “ruffled” the border of the object is. Compatible with our visual inspection of the images, we quantitatively show that differentiating colonies were less circular, with more ruffled borders and increased perimeter size (Figure 4C). A small non-significant increase in colony area was also observed. We thus believe that these features, along with others that may also take into account the pixel intensities within the colonies, may be important for the CNN to be able to display such high predictive power. Of note, all these morphological changes are relatively small, as shown in the density plots of Figure 4C.

### Independent Validation

**Figure 5:**
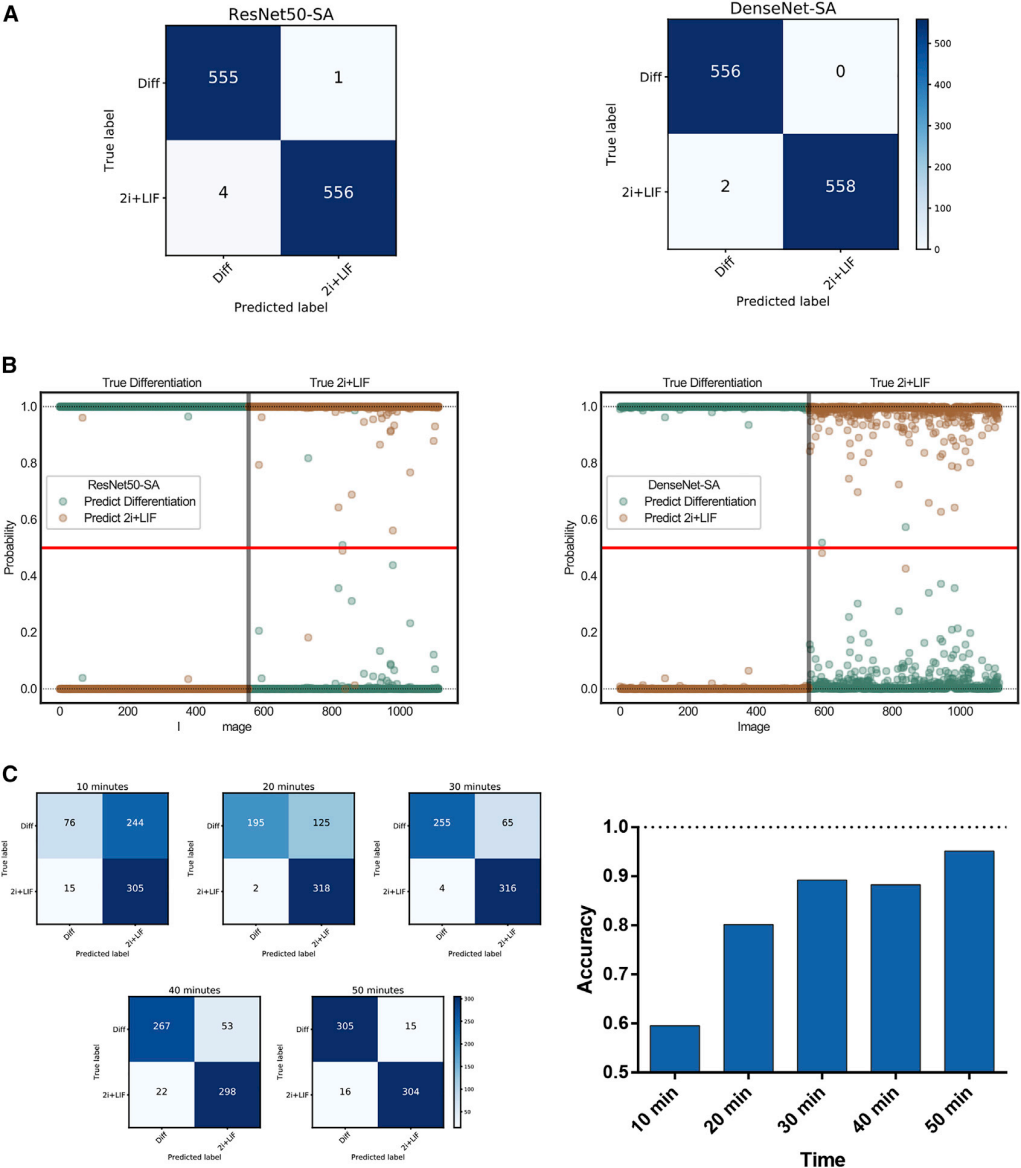
CNN Independent Test (A) Three independent differentiation assays were ran and images were taken as described previously. A total of 1,116 images were analyzed with both ResNet50-SA and DenseNet-SA. Confusion matrices show that both neural networks predicted with high accuracy the differentiating group. (B) Probability plots. For each image, the neural network generates a probability for both 2i + LIF and differentiating groups. Both probabilities sum up to 1. The prediction will be based on the highest probability. Hence, all predictions are above the red horizontal lines. On the left panel, true differentiating images are represented. Except for one image in ResNet50-SA, all predictions were correct. Moreover, probabilities were very high in almost all cases. Predictions were also very high on the right panel (2i + LIF), although more variability is observed, particularly with DenseNet. (C) Evaluation of classification accuracy during the first hour of differentiation. Differentiation was performed as previously indicated, and images were taken every 10 min during the first hour of differentiation and compared with the same number of images in the 2i + LIF condition. Images were classified according to the previously obtained ResNet50-SA training for 1 h of differentiation. Left side, confusion matrices for each time point. Right side, classification accuracy. Of note, by 20–30 min most of the differentiation images are correctly classified.

We then analyzed the performance of the networks in independent biological samples. Once trained, a CNN can be easily used for prediction and run on a simple central processing unit (CPU), without the computational requirements of a graphic processor unit (GPU). We independently tested two of the successful networks (Resnet50-SA and DenseNet-SA) in three more mESC differentiation experiments, completely unrelated from the previous ones. In 1,116 images, CNN were highly accurate (Figure 5A). Overall, ResNet50-SA wrongly identified 4 images of 560 as differentiating, when in fact they were in the 2i + LIF group, and one image as pluripotent when in fact it was differentiating. When DenseNet-SA was used, the misidentification was only 2 of 560 in the 2i + LIF group. These independent results confirmed the high accuracy (0.996 for ResNet50-SA and 0.998 for DenseNet-SA) that both models reached in identifying morphological cell changes at a very early stage of differentiation. Table 1 shows the classification report of the prediction of the three replicates. All independent tests showed high precision and recall, with no significant differences between models.

**Table 1:** CNN Performances on Independent Replicates

CNN	Group	Precision	Recall	F1 Score
2^∗^ResNet50-SA	differentiating	0.9938 ± 0.0061	0.9979 ± 0.0037	0.9958 ± 0.0018
	pluripotent stem cell	0.9979 ± 0.0036	0.9938 ± 0.0063	0.9958 ± 0.0018
2^∗^DenseNet-SA	differentiating	0.9965 ± 0.0032	1.000 ± 0.0000	0.9982 ± 0.0016
	pluripotent stem cell	1.000 ± 0.0000	0.9965 ± 0.0032	0.9983 ± 0.0016

When the CNN classifies each image it outputs two probabilities: one for cells in 2i + LIF, and the other for differentiating EpiLCs. Both probabilities sum up to 1, and the call will be for the higher one. To get an insight of the individual probabilities within all the images in the training experiment, we plotted individual probabilities for both networks (Figure 5B). Both CNNs can easily identify differentiating cells, with a very high probability for each image. All of them, except for a few, are extremely close to 1. However, probabilities for identifying 2i + LIF cells were less high in both CNNs. We think that this is because CNN performs image recognition by identifying object borders. The morphological changes of differentiating cells, with protrusions and spindles, may offer an advantage in this case. Finally, the 2i + LIF prediction was significantly more *precise* with ResNet50-SA, based on higher individual probabilities assigned for each image (mean probability values for the 2i + LIF groups: ResNet50-SA, 0.989 ± 0.078; DenseNet-SA, 0.976 ± 0.055; p < 0.001 by Wilcoxon test). A possible inference from these results is that ResNet50-SA is able to extract more features than DenseNet-SA.

We then wondered if the neural network would be able to correctly classify differentiation at earlier time points. We calculated the classification accuracy on images taken every 10 min from the onset of differentiation, but without re-training the network, i.e., using the 1-h training. We found that the accuracy in these earlier points was still high, reaching a value higher than 0.8 at 20 min. As expected, at earlier time points the CNN tended to classify differentiating cells as being in the 2i + LIF category possibly because colonies did not yet acquire morphological differences. For this reason, the recall of the classification for differentiating images and the precision of classification of 2i + LIF images, respectively, increased with time (Figure 4C, see Figure S7). Video S1 shows the progressive flattening and morphological changes in the cell colonies. These changes are observed as soon as 10 to 20 min from the differentiation stimulus. Of note, we cannot rule out that accuracy would be higher if prediction were based on a neural network trained at earlier time points.

Video S1. Time Lapse Video Showing 46C mESCs Cultured in 2i + LIF and during the First Hour after EpiLC Induction, Related to Figure 5

### CNN Training on Different PSC Experimental Setups

We previously showed that CNNs can be efficiently trained to identify early stages of mESCs differentiation toward EpiLCs. We next decided to explore the applicability of DL into the analysis of morphological differences of PSCs in other experimental setups.

**Figure 6:**
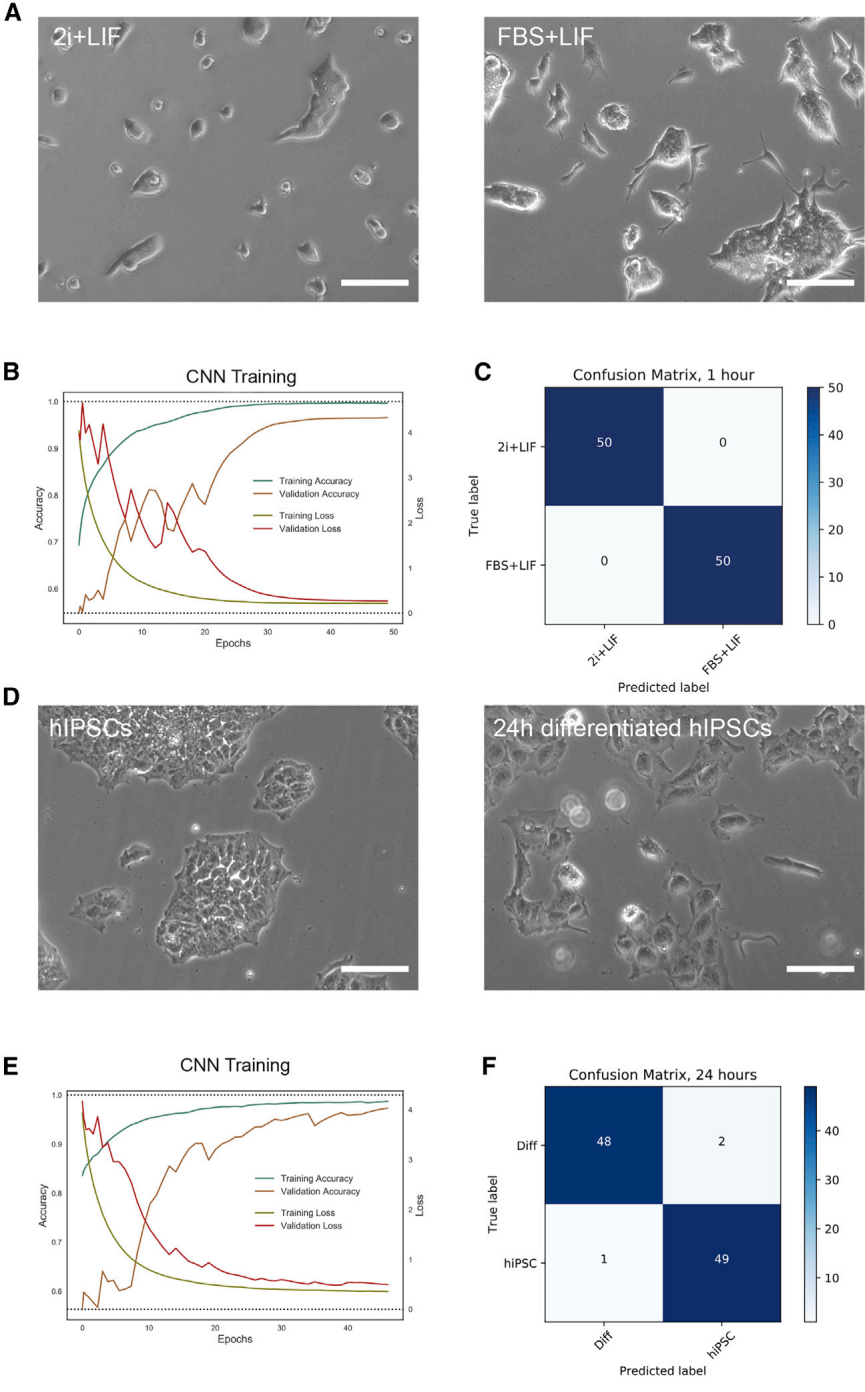
CNN Training on Different PSCs Experimental Setups (A) CNN training and validation of 46C mESCs cultured in 2i + LIF and in FBS + LIF conditions. Representative images in 2i + LIF (left) or FBS + LIF (right). (B) Training and validation accuracy and training and validation loss for mESCs in 2i + LIF versus FBS + LIF. Of note, validation accuracy was above 0.95. (C) Confusion matrix of an independent set of images classified using the trained CNN for mESCs in 2i + LIF versus FBS + LIF. Classification accuracy was of 1. (D) CNN training and validation of undifferentiated hiPSCs and during early mesodermal induction. Representative images of hiPSCs colonies cultured in the undifferentiated state (left) or after 24 h induction in StemPro-34 with Activin A and BMP4, and VEGF (right). (E) Training and validation accuracy and training and validation loss for hiPSCs. Of note, validation accuracy was above 0.95. (F) Confusion matrix of an independent set of images classified using the trained CNN for hiPSCs. Classification accuracy was of 0.97. Scale bars, 100 μm.

First, we assessed whether it was possible to train a CNN to classify mESCs cultured in different conditions. As we previously mentioned, mESCs can be maintained in the ground state of pluripotency when cultured in defined media in the presence of LIF and inhibitors of the MEK/Erk and GSK3 differentiation pathways (2i + LIF) (Ying et al., 2008). Up until the development of these defined conditions, mESCs were routinely cultured in FBS-containing medium in the presence of LIF alone (FBS + LIF), where they remain in a naive pluripotent state but display high population heterogeneity and increased expression of differentiation markers, among other differences (Guo et al., 2016). Interestingly, mESCs in these two naive supporting conditions also display morphological differences. We thus assessed if it was possible to train a CNN to identify the culture condition, and found that the trained CNN reached a very high level of accuracy in predicting which medium was used (Figures 6A–6C).

Finally, we decided to analyze whether a CNN was capable of identifying a completely different type of PSCs and an associated differentiated cell type. As we previously mentioned, terminally differentiated cells can be reprogrammed into an iPSC, which holds great promise in the field of regenerative medicine. We thus decided to differentiate a previously obtained hiPSC line derived in our lab to an early mesodermal progenitor (Questa et al., 2016). To this end, we cultured the hiPSCs in the presence of Activin A, BMP4, and vascular endothelial growth factor (VEGF) for 24 h (Evseenko et al., 2010), and trained a CNN to classify undifferentiated and early mesodermal progenitors. Again, training images using Resnet50 resulted in a very high level of accuracy of classification of independent images (Figures 6D–6F). All these data confirm the high capability of CNNs to identify minor, early changes in stem cell differentiation irrespective of the protocol or cell used.

## Discussion

In this paper we show that current deep CNNs can be trained with a relatively large series of images taken in a simple transmitted light microscope and then correctly classify images with minor morphological changes in independent, new samples. Close to 100% accuracy was reached in most cases. The neural networks demonstrated to be very sensitive to the morphological changes: in a model of mouse PSCs, only 20 to 40 min after the onset of differentiation the CNN demonstrated detection of morphological changes in most of the images. We also demonstrated its efficacy in several settings of pluripotent stem cell culture, including EpiLC differentiation in other mouse PSCs and in early mesoderm differentiation of hiPSCs. Time-lapse imaging shows that these changes are readily observed by the human eye. However, changes are minimum, and entails subtle variations in the cell surface. At these early time points and with the proper cell imaging settings, CNNs were able to at least emulate human visual recognition. We did not compare these results with human prediction. We think this would be misleading, since humans are not necessarily trained to detect such minimal changes, and if they were, we believe that they would or should recognize morphological changes as effectively as the CNN. There are other advantages of a neural network applied to cell models, such as continuous, automatic, real-time detection with high precision. Altogether, such powerful systems will soon overcome human capacity.

The application of DL in this work should be emphasized by its simplicity. We used plain, phase contrast images taken in a transmitted light microscope with a 10× objective. There was no need to process the images in any form or to apply complex protocols for differentiation. Moreover, detection of the morphological changes was performed at a very short time from the beginning of the assay. Training a network has also become simpler with the development of frontend software applications, such as Keras or pyTorch. Finally, the use of GPU allows to process many images with good definition in a relatively short time. Without GPU support, in fact, this training would not have been possible in a sensible amount of time. All these factors make CNNs a field where many image applications will based their analyses in the oncoming years.

We believe that several conditions allowed us to reach such a high accuracy with the trained neural network. First, cell seeding at a high density was important to provide enough information to the algorithm. We cannot rule out, however, that with more training and different set up a CNN would get a high accuracy with just one cell colony. Second, the size of the starting images were 480 × 640 pixels, increasing the calculation burden but providing enough details to the CNN. Third, we trained very deep CNNs, with dozens of hidden layers. A shallower network proved useless to train our set of images. Fourth, we made use of image preprocessing, which artificially increases the number of images provided to the CNNs. We found, however, that too much image preprocessing was detrimental for the accuracy and loss. Hence, most effective trainings were reached when image preprocessing was limited to flipping the image in both directions. However, we also divided each original image in four, which may be seen as zooming into the four quadrants. We believe that subtle image augmentation, such as blurring, contrast, or bright enhancements, could eventually improve performance in other settings, but more technical work is needed to confirm this.

DL predictions applied on a live imaging setup will be one of the most exciting applications in the next few years. Therefore, we were interested in how the trained network would work on images taken at earlier times. We found that the high accuracy starts approximately 30 min from the onset of differentiation, although a moderate accuracy is already seen at 20 min. This experiment predicts the future use of neural networks on real-time prediction in cell culture experiments. Generalization on each specific context will be critical for the applicability of DL techniques.

A few papers are now reporting the use of DL training in the field of cell biology. Some papers processing high complex images have been published, and DL has been shown to provide a great advantage in this setting. Hay and Parthasarathy (2018) used a self-developed CNN to identify bacteria in a 3D microscope images, reaching approximately 90% of accuracy. Pärnamaa and Parts (2017) used also a shallow network to identify subcellular structures, with an accuracy of approximately 90%. Eulenberg et al. (2017) identified the cell-cycle phases in Jurkat cells with high accuracy. However, not too many papers have tried to classify cells based on simple images taken in a transmission light microscope. Recently, Kusumoto et al. (2018) used DL to classify PSC versus PSC-derived endothelial cells after 6 days of differentiation. These authors used two shallow CNNs, LeNet, and AlexNet. These network yielded between 80% and 90% of accuracy in positive identification of the cell population. Although encouraging, these results were far from optimal. The use of these shallow networks may be appealing because of lower computational needs, but they proved not to be accurate compared with the deeper networks used in our paper.

Even though these results are at the top of the possible accuracy, some caveats should be mentioned. First, we applied these CNNs for a limited type of stem cell differentiation assays. To what extent these results translate to other setting remains to be established. We proved an internal validity of the trained network by applying it multiple times, but an external validation (other cells, labs, and/or microscope) remains to be assessed. However, we think that the ability of CNNs is such that it should be able to classify cell images in many different contexts. Second, the field is growing fast. There are many other CNN architectures and strategies (e.g., GAN, segmentation, CapsNets) that deserve attention. We did not try any of them as we got excellent results with our strategies. However, it may be possible to apply them and achieve a better performance, such as reducing training time or reducing the number of images needed to train. Finally, although effective, we kept our work simple. We only compared two groups using a 10× objective and we did not use any fluorescence labeling. Any modification of our experimental setting should be extensively tested, but we believe that the strength of the application of neural networks for image recognition in this setting is proved.

In conclusion, we trained a CNN to identify PSCs from very early differentiating PSCs. The trained network allowed a very high rate of prediction, almost to 1. Moreover, the ability to differentiate may be as low as 20 min after the onset of differentiation. It is hard to think of any other cell assay that can confirm differentiation in such a short time with such precision and at such a low cost. We believe that DL and convoluted neural networks will change how cell assays are performed in the near future.

## Experimental Procedures

### Cells and Differentiation Protocols

Mouse ESCs were grown in defined conditions that support the ground state of pluripotency. In brief, 46C mESCs were grown in the chemically defined medium N2B27 supplemented with 1,000 U/mL human LIF (Gibco), 1 μM PD0325901 (Tocris), and 3 μM CHIR99021 (Tocris), hereafter called “2i + LIF medium.” N2B27 medium formulation is described in detail elsewhere (Waisman et al., 2017). Cells were grown at 37°C in a 5% CO_2_ incubator on 0.1% gelatin-coated dishes and were passaged every 2–3 days using TrypLE (Gibco). To induce EpiLC differentiation, mESCs were plated the day before in 2i + LIF medium at a density of 30 × 10 or 60 × 10 cells/cm. The following day, cells were washed two times with 1× PBS and differentiated in N2B27 medium containing 1% KSR (Gibco), 12 ng/mL basic fibroblast growth factor (Thermo Fisher Scientific), and 20 ng/mL Activin (Thermo Fisher Scientific), hereafter called “EpiLCs medium.” For control cells, fresh 2i + LIF medium was added. To analyze the morphology of cells in the presence of FBS and LIF, cells were grown in DMEM supplemented with 15% FBS, 100 mM minimum essential medium nonessential amino acids, 0.5 mM 2-mercaptoethanol, 2 mM GlutaMax with the addition of 1,000 U/mL of LIF, all reagents purchased from Gibco. Cells were seeded at 60 × 10 cells/cm. The 46C cell line used throughout this work was a kind gift of Austin Smith. E14-derived Ainv15 mESCs used in Figure S2, purchased from ATCC, were also seeded at 60 × 10 cells/cm.

Human induced PSCs were generated previously in our lab (Questa et al., 2016). We regularly grow them in E8-Flex in Geltrex or Vitronectin-coated plates (all Thermo Fischer Scientific). For early mesoderm differentiation, we replaced E8-Flex medium with StemPro-34 medium supplemented with BMP4, Activin A, and VEGF (all 10 ng/μL) for 24 h (Evseenko et al., 2010).

### Cell Imaging and Image Processing

Random images were taken at consecutive hours post differentiation in an EVOS microscope (Thermo Fischer Scientific). Cells were plated at the indicated cell densities in 12-well plates (Corning), and cells were seeded approximately 24 h before imaging. We used a 10× objective with light transmission. Light intensity was set at 40%. Image files were saved in jpg format. The standard output of the EVOS images is 960 × 1,280 pixels in three channels (RGB). Each picture was then sliced to get images of 480 × 640 pixels by applying the python script ImageSlicer (Dobson, 2018). These dimensions were downsized to 240 × 320 at the time of training.

For the images taken in the 24-h experiment, we took images from three biological replicates with two identical wells in each condition, running control and differentiation in parallel. The final number of images was between 300 and 400. For the experiments with 1 and 2 h differentiation, 4 biological replicates were done and between 70 and 100 images were taken from each condition. We then fed the network with 2,134 images for training (900 in the 2i + LIF group and 1,134 in the Differentiation group), and 400 for validation (200 in each group). One hundred images (50 per group) were reserved for independent prediction after training. Independent replicates (n = 3) were run and prepared in the same way.

### Cell Staining and Analysis

For immunofluorescence experiments, cells were grown on Lab-Tek 8-well chamber slide (Nunc) previously coated for 30 min with Geltrex (Thermo Fisher Scientific). Cells were fixed for 20 min with 4% paraformaldehyde, permeabilized with 0.1% Triton X-100 PBS (PBST) and blocked with 3% normal donkey serum in PBST. Primary antibody against phospho-p44/42 MAPK (Erk1/2) (Cell Signaling no. 4,370) was added in block solution, incubated at 4°C overnight, and then washed three times in PBST for 30 min. Texas Red-X Phalloidin (Molecular Probes), secondary antibody and DAPI were incubated in block solution at room temperature for 30 min. Samples were washed as before, mounted, and imaged on an EVOS fluorescence microscope (Thermo Fisher Scientific).

### Real-Time PCR

Gene expression was analyzed as described previously (Waisman et al., 2017). In brief, total RNA was extracted with TRI Reagent (Sigma Aldrich) following the manufacturer's instructions, treated with DNAse (Thermo Fisher Scientific), and reverse transcribed using MMLV reverse transcriptase (Promega). Quantitative PCR was performed in a StepOne Real-Time PCR system (Applied Biosystems). Gene expression was normalized to the geometrical mean of GAPDH and PGK1 housekeeping genes, data were then log transformed and relativized to the average of the biological replicates for the 2i + LIF condition. Primers sequences were reported previously (Waisman et al., 2017). Statistical significance for qPCR data was analyzed by randomized block design ANOVA. Comparison between means were assessed using Tukey test.

### CNN Networks and Training

Neural network trainings were performed in a p2.xlarge instance from Amazon Web Service (www.aws.amazon.com). This instance provides cloud computing with 4 CPUs, a RAM of 61 Gb, and one NDIVIA K80 GPU. Computing was done in a preconfigure environment for DL based on Ubuntu (v.16.04). Training was performed in Keras (v.2.1.5) (Chollet and others, 2015), with TensorFlow (v.1.6.0) as backend. A code example is available in GitHub. Detailed information about CNN training can be found in the Supplemental Experimental Procedures.

### Colony Morphological Analysis

Morphological analyses of cell colonies were performed using FIJI/ImageJ and custom R scripts. For more information, see the Supplemental Experimental Procedures.

## Author Contributions

A.W., A.L.G., and S.G.M. conceived the experiments. A.W., A.L.G., A.M.B., G.N., A.S., and N.L.S.V. performed the cell experiments. A.W. performed the cell morphology analysis. A.W., M.A.S., and A.M.M. performed the gene expression experiments. A.W., L.N.M., C.L., G.L.S., and A.S.G. discussed the experiments and manuscript. G.E.S. and S.G.M. provided the funding for this paper. S.G.M. performed deep learning training and wrote the manuscript.

## Supplemental Information

Document S1. Supplemental Experimental Procedures, Figures S1–S7, and Table S1

Data S1. ResNet50 Model, Related to Figure 2

Data S2. DenseNet Model, Related to Figure 2

Document S2. Article plus Supplemental Information
