# Supplementary material for: Deep Learning Neural Networks Highly Predict Very Early Onset of Pluripotent Stem Cell Differentiation
Source: Stem Cell Reports. 2019 Mar 14;12(4):845–59. doi: 10.1016/j.stemcr.2019.02.004 (PMC6449871; doi:10.1016/j.stemcr.2019.02.004)
Supplement: Document S1. Supplemental Experimental Procedures, Figures S1–S7, and Table S1 [file mmc1.pdf]

Stem Cell Reports, Volume *12*

Supplemental Information

Deep Learning Neural Networks Highly Predict Very Early Onset of Pluripotent Stem Cell Differentiation

Ariel Waisman, Alejandro La Greca, Alan M. Möbbs, María Agustina Scarafía, Natalia L. Santín Velazque, Gabriel Neiman, Lucía N. Moro, Carlos Luzzani, Gustavo E. Sevlever, Alejandra S. Guberman, and Santiago G. Miriuka

Stem Cell Reports

Supplemental Information

Deep learning neural networks highly predict very early onset of pluripotent stem cell differentiation

Ariel Waisman, Alejandro La Greca, Alan M. Möbbs, María Agustina Scarafía, Natalia L. Santín Velazque, Gabriel Neiman, Lucía N. Moro, Carlos Luzzani, Gustavo E. Sevlever, Alejandra S. Guberman, Santiago G. Miriuka

Supplemental Figures

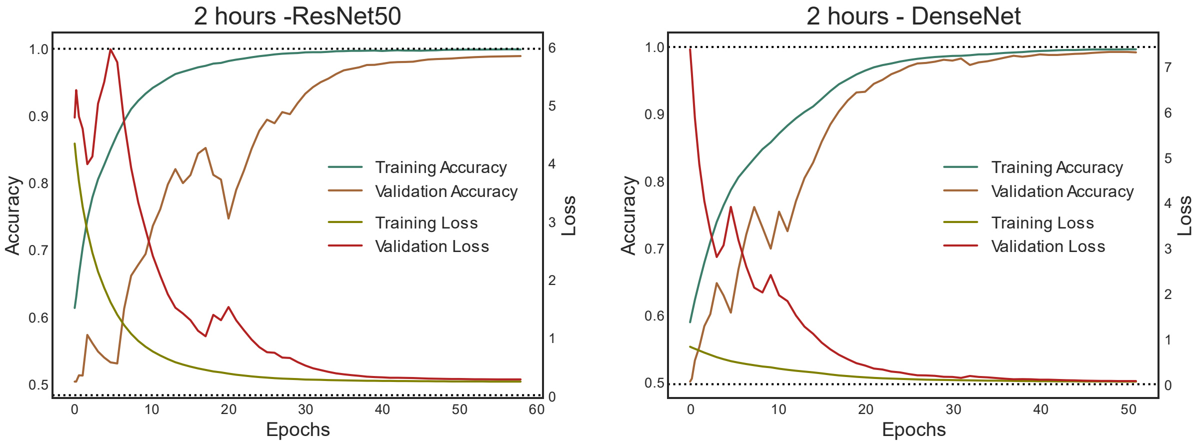

Figure S1: CNN training at 2 hours from onset of differentiation. ***Related to Figure 2*** Images taken after two hours of differentiation were trained with Resnet50 (on the left panel) and DenseNet (on the right panel), in both cases using simple augmentation. With both networks training reached a very high accuracy.

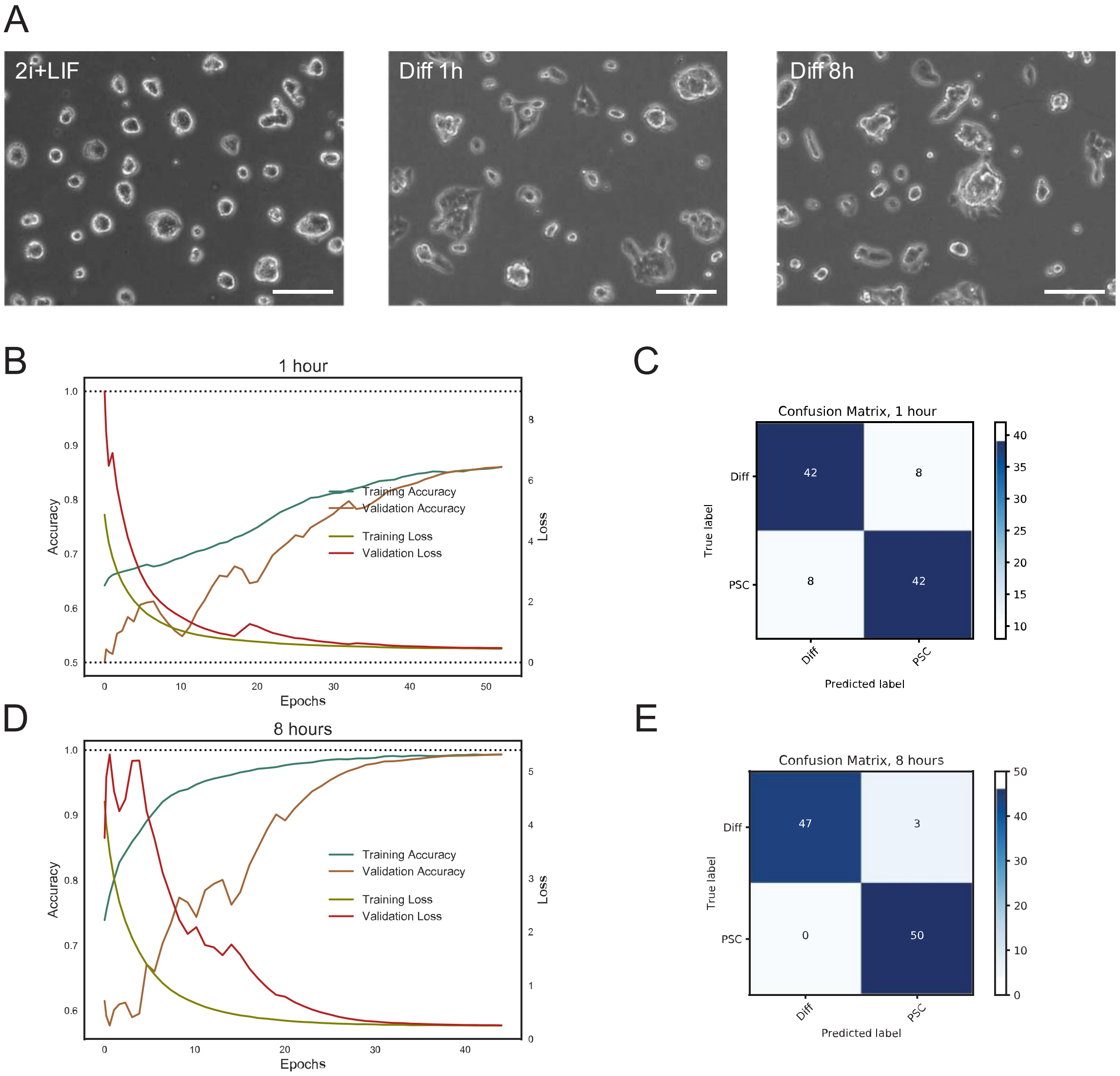

Figure S2: CNN training o f Ainv15 mESCs and after 1 or 8 hours of EpiLC induction. ***Related to Figure 1.*** A. Representative images of Ainv15 mESCs colonies cultured in 2i+LIF (left) or induced to differentiate to EpiLCs for 1h (center) and 8h (right). Bar, 100µm. B. Training and validation accuracy and training and validation loss comparing 2i+LIF and 1h Diff. C. Confusion matrix of an independent set of images classified using the trained CNN for 2i+LIF and 1h Diff training. Classification accuracy was approximately 0.85. D. Training and validation accuracy and training and validation loss comparing 2i+LIF and 8h Diff. E. Confusion matrix of an independent set of images classified using the trained CNN for 2i+LIF and 8h Diff training. Classification accuracy was approximately 0.97.

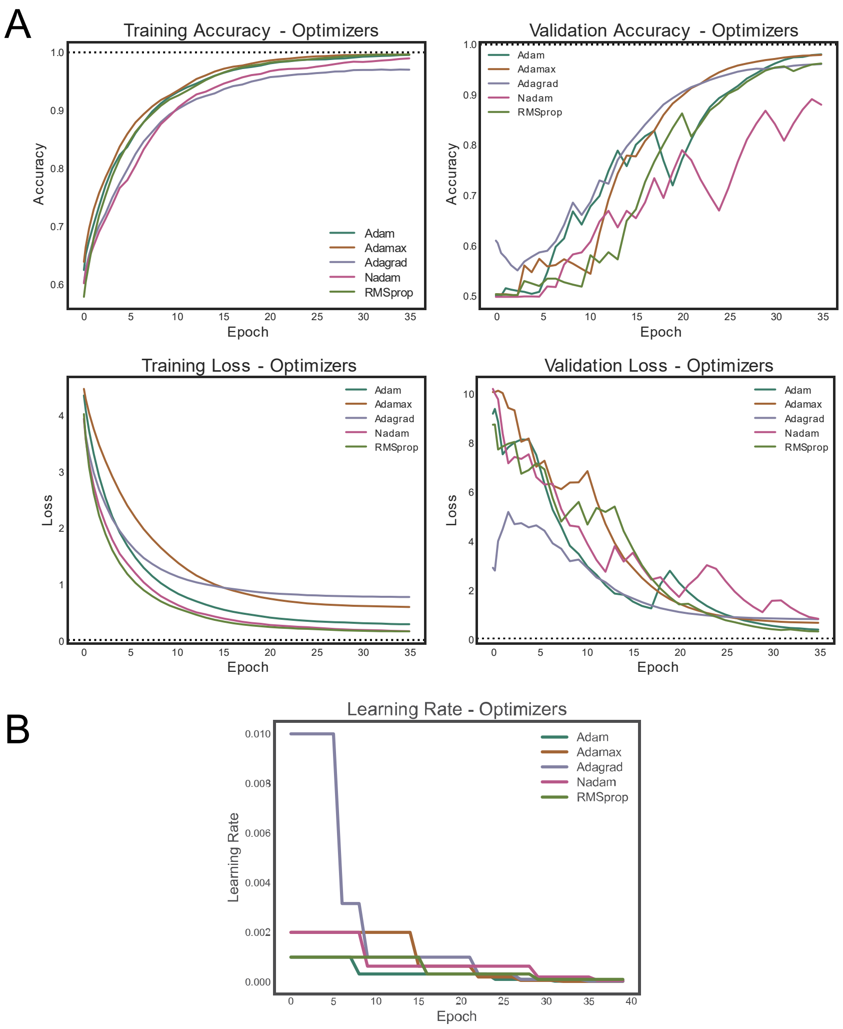

Figure S3: Optimizer analysis. ***Related to Figure 2.*** A. Several algorithms were tested in a short fixed ResNet50 run on the 1 hour images. Although training accuracy behaves similar between groups, validation accuracy was reached faster with Adam, Adagrad, and Adamx. We also tested SGD, but we were unable to train the network with it. B. Learning rate progress through epochs. Each of the optimizers tested decrease overtime to better adjust for finding the minimal optimal loss. All of them progress to minimal learning rate and minimal variations.

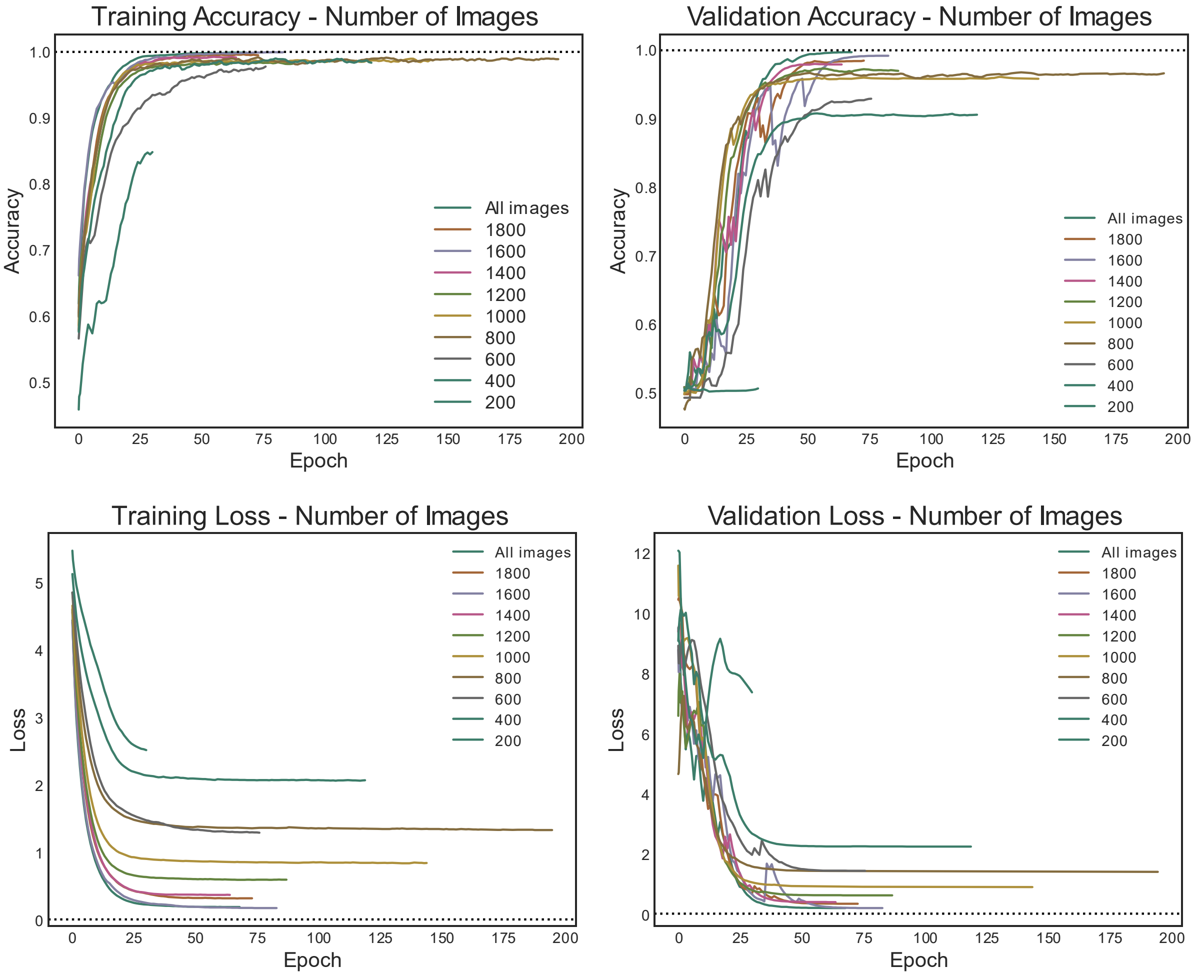

Figure S4: Training according to the number of figures fed to the CNN. ***Related to Figure 2.*** We train ResNet50-SA with progressively lower number of images. All parameters show a loss of training efficiency as the number of images decrease. Underfitting is observed with 1400 images or less, although still a high accuracy is seen even with 800 images. The figure suggests that as more images are provided, the better the neural network trains.

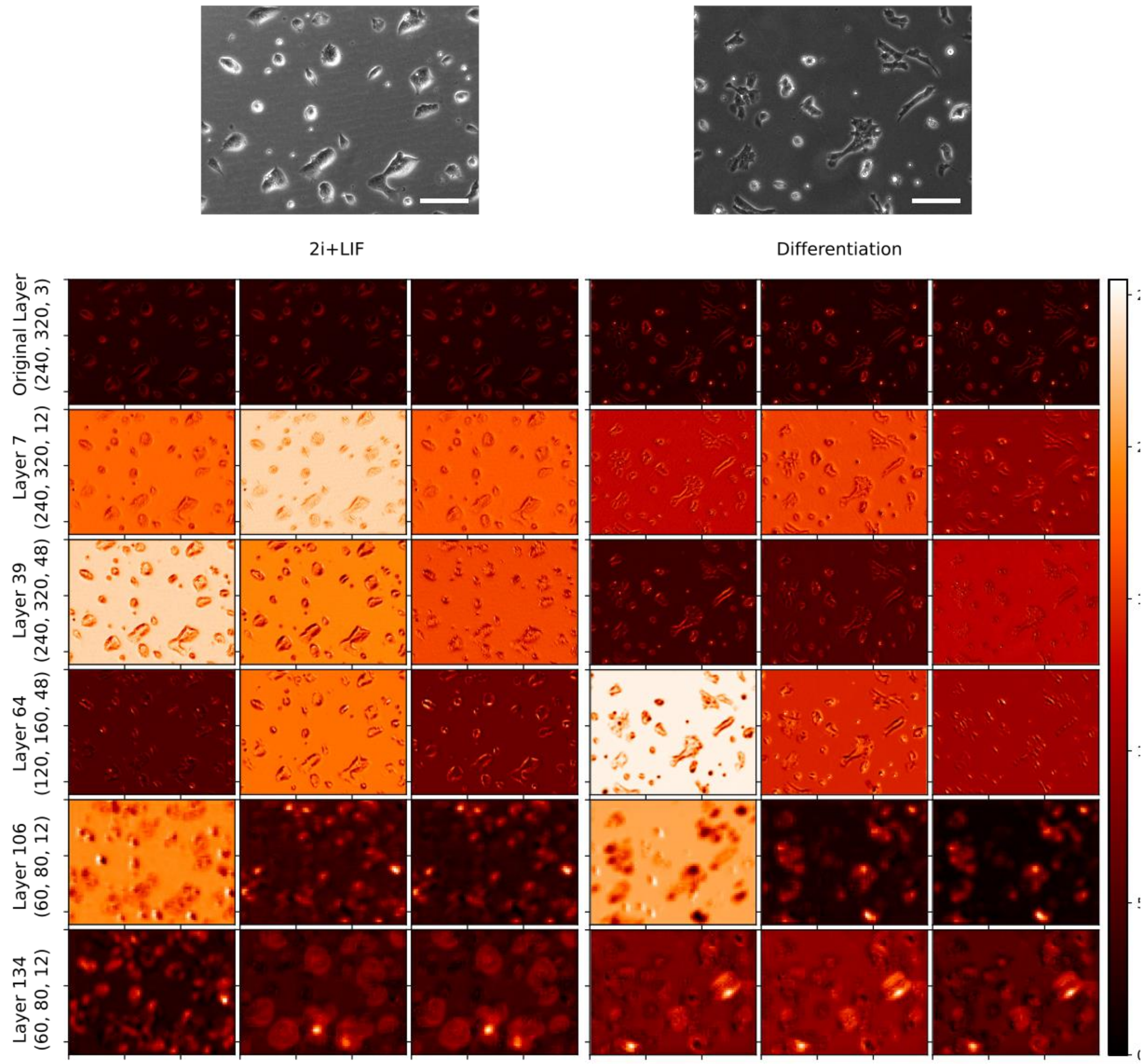

Figure S5: Activation layers in DenseNet. ***Related to Figure 3.*** DenseNet architecture is different than ResNet. Instead of ending in low resolution images with high depth, it ends in a relatively higher resolution image with low depth. However, the representation if the activation in the last row shows that the relationship between activations looks different. In PSC, activations still conserved roundness, as opposed to activations in differentiating cells. Bar, 100 µm.

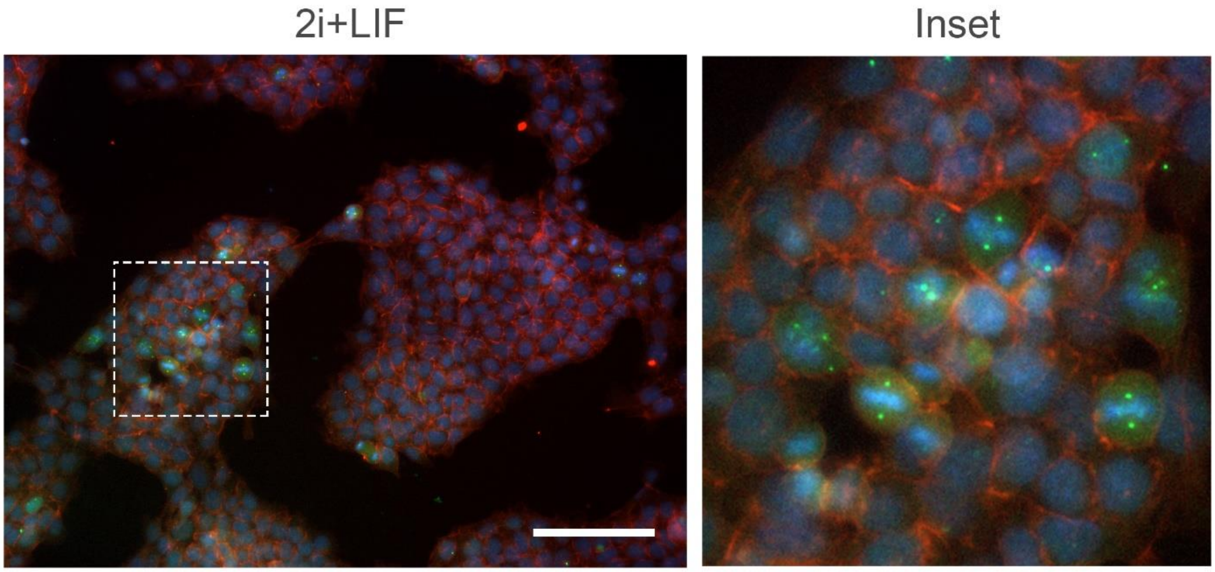

Figure S6: Phospho-Erk1/2 in 2i+LIF cultured mESCs is only present in mitotic cells. ***Related to Figure 4.*** Representative immunostainings of 46C mESCs cultured in 2i+LIF. Cells were evaluated for ERK1/2 phosphorylation (green) and for the re-organization of the actin cytoskeleton (phalloidin, red). Nuclei were stained with DAPI (blue). Bar, 100 µm. The inset shows a higher magnification of mitotic cells, as evaluated by the presence of metaphase chromosomes. Of note, only mitotic cells display high levels of phospho-ERK1/2 in the cytoplasm and as discrete foci in mitotic spindle poles, as previously reported in other cell systems (Shapiro et al., 1998).

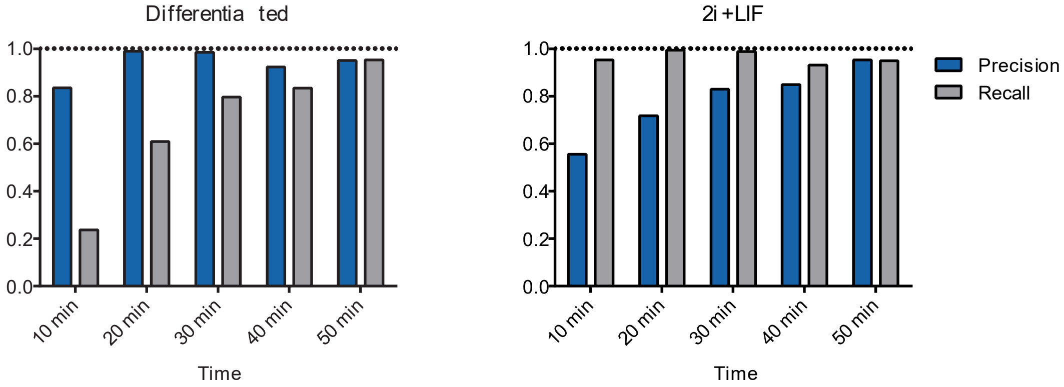

Figure S7: Precision and recall for differentiating 46C mESCs during the first 50 minutes after differentiation stimuli. *Related to Figure 5.* Precision and recall were calculated from the confusion matrix in Figure 5C as described in the Materials and Methods section. Values were calculated considering differentiating images as the true value (left chart) and considering 2i+LIF as the true value (right panel). When evaluating Diff as being the true value, at 10 minutes, the majority of images corresponding to the differentiation class are incorrectly classified as being in the 2i+LIF category, thus the recall is low. However, there are few false positives, i.e., cells truly in 2i+LIF that are incorrectly classified as in the Diff class. Thus, the precision is high. As time progresses, the value of recall increases to almost 1, while the precision is kept constant. On the other hand, when considering 2i+LIF as the true value, at 10 minutes there are few false negative images, i.e., true 2i+LIF images incorrecly classified as Diff, thus the recall is high and is kept constant for the other time points. However, the amount of false positives is initially high and thus the precision is low, which is increased as time progresses.

Video. *Related to Figure 5.* Time lapse video showing 46C mESCs cultured in 2i+LIF and during the first hour after EpiLC induction.

CNN	Training Time	Epochs	Training Accuracy	Validation Accuracy	Training Loss	Validation Loss	Precision
ResNet34 (NIA)	108 min	53	.9970	.9922	.1853	.2002	.98
**ResNet50 (NIA)**	**114 min**	**58**	**.9951**	**.9990**	**.1980**	**.2154**	**1**
ResNet101 (NIA)	219 min	78	1.0000	.9922	.2092	.2314	.99
**ResNet50(SA)**	**130 min**	**73**	**.9990**	**.9974**	**.1839**	**.1889**	**1**
ResNet50 (CA)	150 min	65	.9921	.9740	.1666	.1944	.98
DenseNet (NIA)	305 min	64	.9926	.9850	.0925	.1050	.98
**DesnseNet (SA)**	**279 min**	**57**	**.9916**	**.9950**	**.0789**	**.0783**	**1**

DesnseNet (CA)	216 min	40	.9592	.9575	.1893	.2138	.97

Table S1: Performance measurement on CNNs trained at 1 hour.

Supplemental Experimental Procedures

CNN networks and training

We used previously developed deep convoluted neural networks for training images, including ResNet (Huang et al., 2016) and DenseNet (He et al., 2015). Both were adapted from GitHub repositories. VGG16 was used from Keras. Learning rate adjustment was done with Adam (*adaptive moment estimation*) (Kingma and Ba, 2014), unless specify. We specified when image augmentation was used. We called simple image augmentation (SA) when only flipping in both direction was applied. We called complex image augmentation (CA) when other features (shearing, rotation, whitening, etc) were added to flipping.

CNN training was set to 200 epochs, although we applied an earlier stopping function to avoid unnecessary training and overfitting. In most trainings this early stopping rule applied when reaching long training stability. Network initial performance was assessed by training and validation accuracy, as well as training and validation loss. Based on the true positive (TP) and false positives (FP) identifications, accuracy was calculated as:

$$Accuracy=\frac{TP_{Diff}+TP_{2i+LIF}}{TP_{Diff}+FP_{Diff}+TP_{2i+LIF}+FP_{2i+LIF}} \quad (1)$$

Testing accuracy was determined in an independent set of 100 images and reported as a confusion matrix with the absolute numbers of prediction, and reporting precision, which was calculated for each group as:

$$Precision=\frac{TP}{TP+FP} \quad (2)$$

A final fully independent validation was done in newly collected images. Three independent replicates were performed. A total of 1116 images were analyzed. Prediction was again reported as confusion matrix with absolute numbers, precision, and recall anf F1, which were calculated for each group as:

$$Recall=\frac{TP}{TP+FN} \quad (3)$$

$$F1=2x\,\frac{Precision+Recall}{Precision+Recall} \quad (4)$$

Colony morphological analysis

To analyze the morphological properties of colonies in 2i+LIF or after 1 hour of EpiLC induction, 20 full size images were processed using Ilastik software (Sommer et al., 2011). Binary masks were automatically generated using the pixel classification routine, and then exported and further processed in Fiji/ImageJ. Cell colonies and its morphological properties were automatically quantified and analyzed using custom R scripts. Statistical differences between the populations were assessed using the non-parametric Kolmogorov Smirnov and the Wilcoxon tests.

Primer sequences

Quantitative PCR primers		
**Name**	**Note**	**Sequence (5’-3’)**
**Dnmt3A**	Forward	TGTGGGAGCCTCAATGTCAC
	Reverse	CCTGCAGCAGTTGTTGTTCC
**Esrrb**	Forward	GAACACTCTCGCCTGGTAGG
	Reverse	CGCCTCCAGGTTCTCAATGT
**FGF5**	Forward	CTTCTGCCTCCTCACCAGTC
	Reverse	TCTGCAGATGGAAACCGATG
**GAPDH**	Forward	TGCCAAGGCTGTGGGCAAGG
	Reverse	CGAAGGTGGAAGAGTGGG
**Klf4**	Forward	TACCCTCCTTTCCTGCCAGA
	Reverse	TTTGCCACAGCCTGCATAGT
**Nanog**	Forward	AGGGTCTGCTACTGAGATGCTCTG
	Reverse	CAACCACTGGTTTTTCTGCCACCG
**Oct6**	Forward	CTCACCTTTTCTCCGGGCTT
	Reverse	ATACACAGATGCGGCTCTCG
**Otx2**	Forward	ACCCGGTACCCAGACATCTT
	Reverse	TTCTTGGCAGGCCTCACTTT
**PGK1**	Forward	TGGGCAAGGATGTTCTGTTC
	Reverse	TGCAGTCCCAAAAGCATCAT
**Tbx3**	Forward	GTCTCCATCGTGGGGACATC
	Reverse	GGCCGTAGTGGTGGAAATCT

Supplemental References

He, K., Zhang, X., Ren, S., and Sun, J. (2015). Deep Residual Learning for Image Recognition. ArXiv E-Prints.

Huang, G., Liu, Z., van der Maaten, L., and Weinberger, K. ~Q. (2016). Densely Connected Convolutional Networks. ArXiv E-Prints.

Kingma, D. ~P., and Ba, J. (2014). Adam: A Method for Stochastic Optimization. ArXiv E-Prints.

Shapiro, P.S., Vaisberg, E., Hunt, A.J., Tolwinski, N.S., Whalen, A.M., McIntosh, J.R., and Ahn, N.G. (1998). Activation of the MKK/ERK pathway during somatic cell mitosis: Direct interactions of active ERK with kinetochores and regulation of the mitotic 3F3/2 phosphoantigen. J. Cell Biol. *142*, 1533–1545.

Sommer, C., Straehle, C., Koethe, U., and Hamprecht, F.A. (2011). Ilastik: Interactive learning and segmentation toolkit. In Biomedical Imaging: From Nano to Macro, 2011 IEEE International Symposium On, pp. 230–233.
