# Supplementary material for: Deep Learning Neural Networks Highly Predict Very Early Onset of Pluripotent Stem Cell Differentiation
Source: Stem Cell Reports. 2019 Mar 14;12(4):845–59. doi: 10.1016/j.stemcr.2019.02.004 (PMC6449871; doi:10.1016/j.stemcr.2019.02.004)
Supplement: Data S1. ResNet50 Model, Related to Figure 2 [file mmc2.pdf]

```
ResNet50

__________________________________________________________________________________________________
Layer (type)                    Output Shape         Param #     Connected to
==================================================================================================
input_4 (InputLayer)            (None, 240, 320, 3)  0
__________________________________________________________________________________________________
conv2d_194 (Conv2D)             (None, 120, 160, 64) 9472        input_4[0][0]
__________________________________________________________________________________________________
batch_normalization_183 (BatchN (None, 120, 160, 64) 256         conv2d_194[0][0]
__________________________________________________________________________________________________
activation_183 (Activation)     (None, 120, 160, 64) 0           batch_normalization_183[0][0]
__________________________________________________________________________________________________
max_pooling2d_4 (MaxPooling2D)  (None, 60, 80, 64)   0           activation_183[0][0]
__________________________________________________________________________________________________
conv2d_195 (Conv2D)             (None, 60, 80, 64)   4160        max_pooling2d_4[0][0]
__________________________________________________________________________________________________
batch_normalization_184 (BatchN (None, 60, 80, 64)   256         conv2d_195[0][0]
__________________________________________________________________________________________________
activation_184 (Activation)     (None, 60, 80, 64)   0           batch_normalization_184[0][0]
__________________________________________________________________________________________________
conv2d_196 (Conv2D)             (None, 60, 80, 64)   36928       activation_184[0][0]
__________________________________________________________________________________________________
batch_normalization_185 (BatchN (None, 60, 80, 64)   256         conv2d_196[0][0]
__________________________________________________________________________________________________
activation_185 (Activation)     (None, 60, 80, 64)   0           batch_normalization_185[0][0]
__________________________________________________________________________________________________
conv2d_198 (Conv2D)             (None, 60, 80, 256)  16640       max_pooling2d_4[0][0]
__________________________________________________________________________________________________
conv2d_197 (Conv2D)             (None, 60, 80, 256)  16640       activation_185[0][0]
__________________________________________________________________________________________________
add_66 (Add)                    (None, 60, 80, 256)  0           conv2d_198[0][0]
                                                                 conv2d_197[0][0]
__________________________________________________________________________________________________
batch_normalization_186 (BatchN (None, 60, 80, 256)  1024        add_66[0][0]
__________________________________________________________________________________________________
activation_186 (Activation)     (None, 60, 80, 256)  0           batch_normalization_186[0][0]
__________________________________________________________________________________________________
conv2d_199 (Conv2D)             (None, 60, 80, 64)   16448       activation_186[0][0]
__________________________________________________________________________________________________
batch_normalization_187 (BatchN (None, 60, 80, 64)   256         conv2d_199[0][0]
__________________________________________________________________________________________________
activation_187 (Activation)     (None, 60, 80, 64)   0           batch_normalization_187[0][0]
__________________________________________________________________________________________________
conv2d_200 (Conv2D)             (None, 60, 80, 64)   36928       activation_187[0][0]
__________________________________________________________________________________________________
batch_normalization_188 (BatchN (None, 60, 80, 64)   256         conv2d_200[0][0]
__________________________________________________________________________________________________
activation_188 (Activation)     (None, 60, 80, 64)   0           batch_normalization_188[0][0]
__________________________________________________________________________________________________
```

conv2d_201 (Conv2D)	(None, 60, 80, 256)	16640	activation_188[0][0]
add_67 (Add)	(None, 60, 80, 256)	0	add_66[0][0]<br>conv2d_201[0][0]
batch_normalization_189 (BatchN	(None, 60, 80, 256)	1024	add_67[0][0]
activation_189 (Activation)	(None, 60, 80, 256)	0	batch_normalization_189[0][0]
conv2d_202 (Conv2D)	(None, 60, 80, 64)	16448	activation_189[0][0]
batch_normalization_190 (BatchN	(None, 60, 80, 64)	256	conv2d_202[0][0]
activation_190 (Activation)	(None, 60, 80, 64)	0	batch_normalization_190[0][0]
conv2d_203 (Conv2D)	(None, 60, 80, 64)	36928	activation_190[0][0]
batch_normalization_191 (BatchN	(None, 60, 80, 64)	256	conv2d_203[0][0]
activation_191 (Activation)	(None, 60, 80, 64)	0	batch_normalization_191[0][0]
conv2d_204 (Conv2D)	(None, 60, 80, 256)	16640	activation_191[0][0]
add_68 (Add)	(None, 60, 80, 256)	0	add_67[0][0]<br>conv2d_204[0][0]
batch_normalization_192 (BatchN	(None, 60, 80, 256)	1024	add_68[0][0]
activation_192 (Activation)	(None, 60, 80, 256)	0	batch_normalization_192[0][0]
conv2d_205 (Conv2D)	(None, 30, 40, 128)	32896	activation_192[0][0]
batch_normalization_193 (BatchN	(None, 30, 40, 128)	512	conv2d_205[0][0]
activation_193 (Activation)	(None, 30, 40, 128)	0	batch_normalization_193[0][0]
conv2d_206 (Conv2D)	(None, 30, 40, 128)	147584	activation_193[0][0]
batch_normalization_194 (BatchN	(None, 30, 40, 128)	512	conv2d_206[0][0]
activation_194 (Activation)	(None, 30, 40, 128)	0	batch_normalization_194[0][0]
conv2d_208 (Conv2D)	(None, 30, 40, 512)	131584	add_68[0][0]
conv2d_207 (Conv2D)	(None, 30, 40, 512)	66048	activation_194[0][0]
add_69 (Add)	(None, 30, 40, 512)	0	conv2d_208[0][0]<br>conv2d_207[0][0]
batch_normalization_195 (BatchN	(None, 30, 40, 512)	2048	add_69[0][0]

```
_
activation_195 (Activation)     (None, 30, 40, 512)  0           batch_normalization_195[0][0]
________________________________________________________________________________________________
_
conv2d_209 (Conv2D)             (None, 30, 40, 128)  65664       activation_195[0][0]
________________________________________________________________________________________________
_
batch_normalization_196 (BatchN (None, 30, 40, 128)  512         conv2d_209[0][0]
________________________________________________________________________________________________
_
activation_196 (Activation)     (None, 30, 40, 128)  0           batch_normalization_196[0][0]
________________________________________________________________________________________________
_
conv2d_210 (Conv2D)             (None, 30, 40, 128)  147584      activation_196[0][0]
________________________________________________________________________________________________
_
batch_normalization_197 (BatchN (None, 30, 40, 128)  512         conv2d_210[0][0]
________________________________________________________________________________________________
_
activation_197 (Activation)     (None, 30, 40, 128)  0           batch_normalization_197[0][0]
________________________________________________________________________________________________
_
conv2d_211 (Conv2D)             (None, 30, 40, 512)  66048       activation_197[0][0]
________________________________________________________________________________________________
_
add_70 (Add)                    (None, 30, 40, 512)  0           add_69[0][0]
                                                                 conv2d_211[0][0]
________________________________________________________________________________________________
_
batch_normalization_198 (BatchN (None, 30, 40, 512)  2048        add_70[0][0]
________________________________________________________________________________________________
_
activation_198 (Activation)     (None, 30, 40, 512)  0           batch_normalization_198[0][0]
________________________________________________________________________________________________
_
conv2d_212 (Conv2D)             (None, 30, 40, 128)  65664       activation_198[0][0]
________________________________________________________________________________________________
_
batch_normalization_199 (BatchN (None, 30, 40, 128)  512         conv2d_212[0][0]
________________________________________________________________________________________________
_
activation_199 (Activation)     (None, 30, 40, 128)  0           batch_normalization_199[0][0]
________________________________________________________________________________________________
_
conv2d_213 (Conv2D)             (None, 30, 40, 128)  147584      activation_199[0][0]
________________________________________________________________________________________________
_
batch_normalization_200 (BatchN (None, 30, 40, 128)  512         conv2d_213[0][0]
________________________________________________________________________________________________
_
activation_200 (Activation)     (None, 30, 40, 128)  0           batch_normalization_200[0][0]
________________________________________________________________________________________________
_
conv2d_214 (Conv2D)             (None, 30, 40, 512)  66048       activation_200[0][0]
________________________________________________________________________________________________
_
add_71 (Add)                    (None, 30, 40, 512)  0           add_70[0][0]
                                                                 conv2d_214[0][0]
________________________________________________________________________________________________
_
batch_normalization_201 (BatchN (None, 30, 40, 512)  2048        add_71[0][0]
________________________________________________________________________________________________
_
activation_201 (Activation)     (None, 30, 40, 512)  0           batch_normalization_201[0][0]
________________________________________________________________________________________________
_
conv2d_215 (Conv2D)             (None, 30, 40, 128)  65664       activation_201[0][0]
________________________________________________________________________________________________
_
batch_normalization_202 (BatchN (None, 30, 40, 128)  512         conv2d_215[0][0]
________________________________________________________________________________________________
_
activation_202 (Activation)     (None, 30, 40, 128)  0           batch_normalization_202[0][0]
________________________________________________________________________________________________
_
```

```
conv2d_216 (Conv2D)             (None, 30, 40, 128)  147584      activation_202[0][0]
________________________________________________________________________________________________
batch_normalization_203 (BatchN (None, 30, 40, 128)  512         conv2d_216[0][0]
________________________________________________________________________________________________
activation_203 (Activation)     (None, 30, 40, 128)  0           batch_normalization_203[0][0]
________________________________________________________________________________________________
conv2d_217 (Conv2D)             (None, 30, 40, 512)  66048       activation_203[0][0]
________________________________________________________________________________________________
add_72 (Add)                    (None, 30, 40, 512)  0           add_71[0][0]
                                                                 conv2d_217[0][0]
________________________________________________________________________________________________
batch_normalization_204 (BatchN (None, 30, 40, 512)  2048        add_72[0][0]
________________________________________________________________________________________________
activation_204 (Activation)     (None, 30, 40, 512)  0           batch_normalization_204[0][0]
________________________________________________________________________________________________
conv2d_218 (Conv2D)             (None, 15, 20, 256)  131328      activation_204[0][0]
________________________________________________________________________________________________
batch_normalization_205 (BatchN (None, 15, 20, 256)  1024        conv2d_218[0][0]
________________________________________________________________________________________________
activation_205 (Activation)     (None, 15, 20, 256)  0           batch_normalization_205[0][0]
________________________________________________________________________________________________
conv2d_219 (Conv2D)             (None, 15, 20, 256)  590080      activation_205[0][0]
________________________________________________________________________________________________
batch_normalization_206 (BatchN (None, 15, 20, 256)  1024        conv2d_219[0][0]
________________________________________________________________________________________________
activation_206 (Activation)     (None, 15, 20, 256)  0           batch_normalization_206[0][0]
________________________________________________________________________________________________
conv2d_221 (Conv2D)             (None, 15, 20, 1024) 525312      add_72[0][0]
________________________________________________________________________________________________
conv2d_220 (Conv2D)             (None, 15, 20, 1024) 263168      activation_206[0][0]
________________________________________________________________________________________________
add_73 (Add)                    (None, 15, 20, 1024) 0           conv2d_221[0][0]
                                                                 conv2d_220[0][0]
________________________________________________________________________________________________
batch_normalization_207 (BatchN (None, 15, 20, 1024) 4096        add_73[0][0]
________________________________________________________________________________________________
activation_207 (Activation)     (None, 15, 20, 1024) 0           batch_normalization_207[0][0]
________________________________________________________________________________________________
conv2d_222 (Conv2D)             (None, 15, 20, 256)  262400      activation_207[0][0]
________________________________________________________________________________________________
batch_normalization_208 (BatchN (None, 15, 20, 256)  1024        conv2d_222[0][0]
________________________________________________________________________________________________
activation_208 (Activation)     (None, 15, 20, 256)  0           batch_normalization_208[0][0]
________________________________________________________________________________________________
conv2d_223 (Conv2D)             (None, 15, 20, 256)  590080      activation_208[0][0]
________________________________________________________________________________________________
batch_normalization_209 (BatchN (None, 15, 20, 256)  1024        conv2d_223[0][0]
________________________________________________________________________________________________
activation_209 (Activation)     (None, 15, 20, 256)  0           batch_normalization_209[0][0]
________________________________________________________________________________________________
conv2d_224 (Conv2D)             (None, 15, 20, 1024) 263168      activation_209[0][0]
```

```
________________________________________________________________________________________________
_
add_74 (Add)                    (None, 15, 20, 1024) 0           add_73[0][0]
                                                                 conv2d_224[0][0]
________________________________________________________________________________________________
_
batch_normalization_210 (BatchN (None, 15, 20, 1024) 4096        add_74[0][0]
________________________________________________________________________________________________
_
activation_210 (Activation)     (None, 15, 20, 1024) 0           batch_normalization_210[0][0]
________________________________________________________________________________________________
_
conv2d_225 (Conv2D)             (None, 15, 20, 256)  262400      activation_210[0][0]
________________________________________________________________________________________________
_
batch_normalization_211 (BatchN (None, 15, 20, 256)  1024        conv2d_225[0][0]
________________________________________________________________________________________________
_
activation_211 (Activation)     (None, 15, 20, 256)  0           batch_normalization_211[0][0]
________________________________________________________________________________________________
_
conv2d_226 (Conv2D)             (None, 15, 20, 256)  590080      activation_211[0][0]
________________________________________________________________________________________________
_
batch_normalization_212 (BatchN (None, 15, 20, 256)  1024        conv2d_226[0][0]
________________________________________________________________________________________________
_
activation_212 (Activation)     (None, 15, 20, 256)  0           batch_normalization_212[0][0]
________________________________________________________________________________________________
_
conv2d_227 (Conv2D)             (None, 15, 20, 1024) 263168      activation_212[0][0]
________________________________________________________________________________________________
_
add_75 (Add)                    (None, 15, 20, 1024) 0           add_74[0][0]
                                                                 conv2d_227[0][0]
________________________________________________________________________________________________
_
batch_normalization_213 (BatchN (None, 15, 20, 1024) 4096        add_75[0][0]
________________________________________________________________________________________________
_
activation_213 (Activation)     (None, 15, 20, 1024) 0           batch_normalization_213[0][0]
________________________________________________________________________________________________
_
conv2d_228 (Conv2D)             (None, 15, 20, 256)  262400      activation_213[0][0]
________________________________________________________________________________________________
_
batch_normalization_214 (BatchN (None, 15, 20, 256)  1024        conv2d_228[0][0]
________________________________________________________________________________________________
_
activation_214 (Activation)     (None, 15, 20, 256)  0           batch_normalization_214[0][0]
________________________________________________________________________________________________
_
conv2d_229 (Conv2D)             (None, 15, 20, 256)  590080      activation_214[0][0]
________________________________________________________________________________________________
_
batch_normalization_215 (BatchN (None, 15, 20, 256)  1024        conv2d_229[0][0]
________________________________________________________________________________________________
_
activation_215 (Activation)     (None, 15, 20, 256)  0           batch_normalization_215[0][0]
________________________________________________________________________________________________
_
conv2d_230 (Conv2D)             (None, 15, 20, 1024) 263168      activation_215[0][0]
________________________________________________________________________________________________
_
add_76 (Add)                    (None, 15, 20, 1024) 0           add_75[0][0]
                                                                 conv2d_230[0][0]
________________________________________________________________________________________________
_
batch_normalization_216 (BatchN (None, 15, 20, 1024) 4096        add_76[0][0]
________________________________________________________________________________________________
_
activation_216 (Activation)     (None, 15, 20, 1024) 0           batch_normalization_216[0][0]
________________________________________________________________________________________________
_
conv2d_231 (Conv2D)             (None, 15, 20, 256)  262400      activation_216[0][0]
```

```
batch_normalization_217 (BatchN (None, 15, 20, 256)  1024        conv2d_231[0][0]

activation_217 (Activation)     (None, 15, 20, 256)  0           batch_normalization_217[0][0]

conv2d_232 (Conv2D)             (None, 15, 20, 256)  590080      activation_217[0][0]

batch_normalization_218 (BatchN (None, 15, 20, 256)  1024        conv2d_232[0][0]

activation_218 (Activation)     (None, 15, 20, 256)  0           batch_normalization_218[0][0]

conv2d_233 (Conv2D)             (None, 15, 20, 1024) 263168      activation_218[0][0]

add_77 (Add)                    (None, 15, 20, 1024) 0           add_76[0][0]
                                                                 conv2d_233[0][0]

batch_normalization_219 (BatchN (None, 15, 20, 1024) 4096        add_77[0][0]

activation_219 (Activation)     (None, 15, 20, 1024) 0           batch_normalization_219[0][0]

conv2d_234 (Conv2D)             (None, 15, 20, 256)  262400      activation_219[0][0]

batch_normalization_220 (BatchN (None, 15, 20, 256)  1024        conv2d_234[0][0]

activation_220 (Activation)     (None, 15, 20, 256)  0           batch_normalization_220[0][0]

conv2d_235 (Conv2D)             (None, 15, 20, 256)  590080      activation_220[0][0]

batch_normalization_221 (BatchN (None, 15, 20, 256)  1024        conv2d_235[0][0]

activation_221 (Activation)     (None, 15, 20, 256)  0           batch_normalization_221[0][0]

conv2d_236 (Conv2D)             (None, 15, 20, 1024) 263168      activation_221[0][0]

add_78 (Add)                    (None, 15, 20, 1024) 0           add_77[0][0]
                                                                 conv2d_236[0][0]

batch_normalization_222 (BatchN (None, 15, 20, 1024) 4096        add_78[0][0]

activation_222 (Activation)     (None, 15, 20, 1024) 0           batch_normalization_222[0][0]

conv2d_237 (Conv2D)             (None, 8, 10, 512)   524800      activation_222[0][0]

batch_normalization_223 (BatchN (None, 8, 10, 512)   2048        conv2d_237[0][0]

activation_223 (Activation)     (None, 8, 10, 512)   0           batch_normalization_223[0][0]

conv2d_238 (Conv2D)             (None, 8, 10, 512)   2359808     activation_223[0][0]

batch_normalization_224 (BatchN (None, 8, 10, 512)   2048        conv2d_238[0][0]
```

activation_224 (Activation)	(None, 8, 10, 512)	0	batch_normalization_224[0][0]
conv2d_240 (Conv2D)	(None, 8, 10, 2048)	2099200	add_78[0][0]
conv2d_239 (Conv2D)	(None, 8, 10, 2048)	1050624	activation_224[0][0]
add_79 (Add)	(None, 8, 10, 2048)	0	conv2d_240[0][0]<br>conv2d_239[0][0]
batch_normalization_225 (BatchN	(None, 8, 10, 2048)	8192	add_79[0][0]
activation_225 (Activation)	(None, 8, 10, 2048)	0	batch_normalization_225[0][0]
conv2d_241 (Conv2D)	(None, 8, 10, 512)	1049088	activation_225[0][0]
batch_normalization_226 (BatchN	(None, 8, 10, 512)	2048	conv2d_241[0][0]
activation_226 (Activation)	(None, 8, 10, 512)	0	batch_normalization_226[0][0]
conv2d_242 (Conv2D)	(None, 8, 10, 512)	2359808	activation_226[0][0]
batch_normalization_227 (BatchN	(None, 8, 10, 512)	2048	conv2d_242[0][0]
activation_227 (Activation)	(None, 8, 10, 512)	0	batch_normalization_227[0][0]
conv2d_243 (Conv2D)	(None, 8, 10, 2048)	1050624	activation_227[0][0]
add_80 (Add)	(None, 8, 10, 2048)	0	add_79[0][0]<br>conv2d_243[0][0]
batch_normalization_228 (BatchN	(None, 8, 10, 2048)	8192	add_80[0][0]
activation_228 (Activation)	(None, 8, 10, 2048)	0	batch_normalization_228[0][0]
conv2d_244 (Conv2D)	(None, 8, 10, 512)	1049088	activation_228[0][0]
batch_normalization_229 (BatchN	(None, 8, 10, 512)	2048	conv2d_244[0][0]
activation_229 (Activation)	(None, 8, 10, 512)	0	batch_normalization_229[0][0]
conv2d_245 (Conv2D)	(None, 8, 10, 512)	2359808	activation_229[0][0]
batch_normalization_230 (BatchN	(None, 8, 10, 512)	2048	conv2d_245[0][0]
activation_230 (Activation)	(None, 8, 10, 512)	0	batch_normalization_230[0][0]
conv2d_246 (Conv2D)	(None, 8, 10, 2048)	1050624	activation_230[0][0]
add_81 (Add)	(None, 8, 10, 2048)	0	add_80[0][0]<br>conv2d_246[0][0]

```
_
batch_normalization_231 (BatchN (None, 8, 10, 2048)  8192        add_81[0][0]
________________________________________________________________________________
_
activation_231 (Activation)     (None, 8, 10, 2048)  0           batch_normalization_231[0][0]
________________________________________________________________________________
_
average_pooling2d_4 (AveragePoo (None, 1, 1, 2048)   0           activation_231[0][0]
________________________________________________________________________________
_
flatten_4 (Flatten)             (None, 2048)         0           average_pooling2d_4[0][0]
________________________________________________________________________________
_
dense_4 (Dense)                 (None, 2)            4098        flatten_4[0][0]
================================================================================
=
Total params: 23,576,450
Trainable params: 23,531,010
Non-trainable params: 45,440
________________________________________________________________________________
_
None
```
