# Supplementary material for: Deep Learning Neural Networks Highly Predict Very Early Onset of Pluripotent Stem Cell Differentiation
Source: Stem Cell Reports. 2019 Mar 14;12(4):845–59. doi: 10.1016/j.stemcr.2019.02.004 (PMC6449871; doi:10.1016/j.stemcr.2019.02.004)
Supplement: Data S2. DenseNet Model, Related to Figure 2 [file mmc3.pdf]

DesneNet

Layer (type)	Output Shape	Param #	Connected to
input_2 (InputLayer)	(None, 240, 320, 3)	0	
conv2d_100 (Conv2D)	(None, 240, 320, 24)	648	input_2[0][0]
batch_normalization_100 (BatchN	(None, 240, 320, 24)	96	conv2d_100[0][0]
activation_100 (Activation)	(None, 240, 320, 24)	0	batch_normalization_100[0][0]
conv2d_101 (Conv2D)	(None, 240, 320, 48)	1152	activation_100[0][0]
batch_normalization_101 (BatchN	(None, 240, 320, 48)	192	conv2d_101[0][0]
activation_101 (Activation)	(None, 240, 320, 48)	0	batch_normalization_101[0][0]
conv2d_102 (Conv2D)	(None, 240, 320, 12)	5184	activation_101[0][0]
concatenate_49 (Concatenate)	(None, 240, 320, 36)	0	conv2d_100[0][0]<br>conv2d_102[0][0]
batch_normalization_102 (BatchN	(None, 240, 320, 36)	144	concatenate_49[0][0]
activation_102 (Activation)	(None, 240, 320, 36)	0	batch_normalization_102[0][0]
conv2d_103 (Conv2D)	(None, 240, 320, 48)	1728	activation_102[0][0]
batch_normalization_103 (BatchN	(None, 240, 320, 48)	192	conv2d_103[0][0]
activation_103 (Activation)	(None, 240, 320, 48)	0	batch_normalization_103[0][0]
conv2d_104 (Conv2D)	(None, 240, 320, 12)	5184	activation_103[0][0]
concatenate_50 (Concatenate)	(None, 240, 320, 48)	0	concatenate_49[0][0]<br>conv2d_104[0][0]
batch_normalization_104 (BatchN	(None, 240, 320, 48)	192	concatenate_50[0][0]
activation_104 (Activation)	(None, 240, 320, 48)	0	batch_normalization_104[0][0]
conv2d_105 (Conv2D)	(None, 240, 320, 48)	2304	activation_104[0][0]
batch_normalization_105 (BatchN	(None, 240, 320, 48)	192	conv2d_105[0][0]
activation_105 (Activation)	(None, 240, 320, 48)	0	batch_normalization_105[0][0]
conv2d_106 (Conv2D)	(None, 240, 320, 12)	5184	activation_105[0][0]

```
________________________________________________________________________________________________
_
concatenate_51 (Concatenate)    (None, 240, 320, 60) 0           concatenate_50[0][0]
                                                                 conv2d_106[0][0]
________________________________________________________________________________________________
_
batch_normalization_106 (BatchN (None, 240, 320, 60) 240         concatenate_51[0][0]
________________________________________________________________________________________________
_
activation_106 (Activation)     (None, 240, 320, 60) 0           batch_normalization_106[0][0]
________________________________________________________________________________________________
_
conv2d_107 (Conv2D)             (None, 240, 320, 48) 2880        activation_106[0][0]
________________________________________________________________________________________________
_
batch_normalization_107 (BatchN (None, 240, 320, 48) 192         conv2d_107[0][0]
________________________________________________________________________________________________
_
activation_107 (Activation)     (None, 240, 320, 48) 0           batch_normalization_107[0][0]
________________________________________________________________________________________________
_
conv2d_108 (Conv2D)             (None, 240, 320, 12) 5184        activation_107[0][0]
________________________________________________________________________________________________
_
concatenate_52 (Concatenate)    (None, 240, 320, 72) 0           concatenate_51[0][0]
                                                                 conv2d_108[0][0]
________________________________________________________________________________________________
_
batch_normalization_108 (BatchN (None, 240, 320, 72) 288         concatenate_52[0][0]
________________________________________________________________________________________________
_
activation_108 (Activation)     (None, 240, 320, 72) 0           batch_normalization_108[0][0]
________________________________________________________________________________________________
_
conv2d_109 (Conv2D)             (None, 240, 320, 48) 3456        activation_108[0][0]
________________________________________________________________________________________________
_
batch_normalization_109 (BatchN (None, 240, 320, 48) 192         conv2d_109[0][0]
________________________________________________________________________________________________
_
activation_109 (Activation)     (None, 240, 320, 48) 0           batch_normalization_109[0][0]
________________________________________________________________________________________________
_
conv2d_110 (Conv2D)             (None, 240, 320, 12) 5184        activation_109[0][0]
________________________________________________________________________________________________
_
concatenate_53 (Concatenate)    (None, 240, 320, 84) 0           concatenate_52[0][0]
                                                                 conv2d_110[0][0]
________________________________________________________________________________________________
_
batch_normalization_110 (BatchN (None, 240, 320, 84) 336         concatenate_53[0][0]
________________________________________________________________________________________________
_
activation_110 (Activation)     (None, 240, 320, 84) 0           batch_normalization_110[0][0]
________________________________________________________________________________________________
_
conv2d_111 (Conv2D)             (None, 240, 320, 48) 4032        activation_110[0][0]
________________________________________________________________________________________________
_
batch_normalization_111 (BatchN (None, 240, 320, 48) 192         conv2d_111[0][0]
________________________________________________________________________________________________
_
activation_111 (Activation)     (None, 240, 320, 48) 0           batch_normalization_111[0][0]
________________________________________________________________________________________________
_
conv2d_112 (Conv2D)             (None, 240, 320, 12) 5184        activation_111[0][0]
________________________________________________________________________________________________
_
concatenate_54 (Concatenate)    (None, 240, 320, 96) 0           concatenate_53[0][0]
                                                                 conv2d_112[0][0]
________________________________________________________________________________________________
_
batch_normalization_112 (BatchN (None, 240, 320, 96) 384         concatenate_54[0][0]
________________________________________________________________________________________________
_
```

```
activation_112 (Activation)     (None, 240, 320, 96) 0           batch_normalization_112[0][0]
__________________________________________________________________________________________________
conv2d_113 (Conv2D)             (None, 240, 320, 48) 4608        activation_112[0][0]
__________________________________________________________________________________________________
average_pooling2d_3 (AveragePoo (None, 120, 160, 48) 0           conv2d_113[0][0]
__________________________________________________________________________________________________
batch_normalization_113 (BatchN (None, 120, 160, 48) 192         average_pooling2d_3[0][0]
__________________________________________________________________________________________________
activation_113 (Activation)     (None, 120, 160, 48) 0           batch_normalization_113[0][0]
__________________________________________________________________________________________________
conv2d_114 (Conv2D)             (None, 120, 160, 48) 2304        activation_113[0][0]
__________________________________________________________________________________________________
batch_normalization_114 (BatchN (None, 120, 160, 48) 192         conv2d_114[0][0]
__________________________________________________________________________________________________
activation_114 (Activation)     (None, 120, 160, 48) 0           batch_normalization_114[0][0]
__________________________________________________________________________________________________
conv2d_115 (Conv2D)             (None, 120, 160, 12) 5184        activation_114[0][0]
__________________________________________________________________________________________________
concatenate_55 (Concatenate)    (None, 120, 160, 60) 0           average_pooling2d_3[0][0]
                                                                 conv2d_115[0][0]
__________________________________________________________________________________________________
batch_normalization_115 (BatchN (None, 120, 160, 60) 240         concatenate_55[0][0]
__________________________________________________________________________________________________
activation_115 (Activation)     (None, 120, 160, 60) 0           batch_normalization_115[0][0]
__________________________________________________________________________________________________
conv2d_116 (Conv2D)             (None, 120, 160, 48) 2880        activation_115[0][0]
__________________________________________________________________________________________________
batch_normalization_116 (BatchN (None, 120, 160, 48) 192         conv2d_116[0][0]
__________________________________________________________________________________________________
activation_116 (Activation)     (None, 120, 160, 48) 0           batch_normalization_116[0][0]
__________________________________________________________________________________________________
conv2d_117 (Conv2D)             (None, 120, 160, 12) 5184        activation_116[0][0]
__________________________________________________________________________________________________
concatenate_56 (Concatenate)    (None, 120, 160, 72) 0           concatenate_55[0][0]
                                                                 conv2d_117[0][0]
__________________________________________________________________________________________________
batch_normalization_117 (BatchN (None, 120, 160, 72) 288         concatenate_56[0][0]
__________________________________________________________________________________________________
activation_117 (Activation)     (None, 120, 160, 72) 0           batch_normalization_117[0][0]
__________________________________________________________________________________________________
conv2d_118 (Conv2D)             (None, 120, 160, 48) 3456        activation_117[0][0]
__________________________________________________________________________________________________
batch_normalization_118 (BatchN (None, 120, 160, 48) 192         conv2d_118[0][0]
__________________________________________________________________________________________________
activation_118 (Activation)     (None, 120, 160, 48) 0           batch_normalization_118[0][0]
__________________________________________________________________________________________________
conv2d_119 (Conv2D)             (None, 120, 160, 12) 5184        activation_118[0][0]
__________________________________________________________________________________________________
concatenate_57 (Concatenate)    (None, 120, 160, 84) 0           concatenate_56[0][0]
                                                                 conv2d_119[0][0]
__________________________________________________________________________________________________
```

```
batch_normalization_119 (BatchN (None, 120, 160, 84) 336         concatenate_57[0][0]
__________________________________________________________________________________________________
_
activation_119 (Activation)     (None, 120, 160, 84) 0           batch_normalization_119[0][0]
__________________________________________________________________________________________________
_
conv2d_120 (Conv2D)             (None, 120, 160, 48) 4032        activation_119[0][0]
__________________________________________________________________________________________________
_
batch_normalization_120 (BatchN (None, 120, 160, 48) 192         conv2d_120[0][0]
__________________________________________________________________________________________________
_
activation_120 (Activation)     (None, 120, 160, 48) 0           batch_normalization_120[0][0]
__________________________________________________________________________________________________
_
conv2d_121 (Conv2D)             (None, 120, 160, 12) 5184        activation_120[0][0]
__________________________________________________________________________________________________
_
concatenate_58 (Concatenate)    (None, 120, 160, 96) 0           concatenate_57[0][0]
                                                                 conv2d_121[0][0]
__________________________________________________________________________________________________
_
batch_normalization_121 (BatchN (None, 120, 160, 96) 384         concatenate_58[0][0]
__________________________________________________________________________________________________
_
activation_121 (Activation)     (None, 120, 160, 96) 0           batch_normalization_121[0][0]
__________________________________________________________________________________________________
_
conv2d_122 (Conv2D)             (None, 120, 160, 48) 4608        activation_121[0][0]
__________________________________________________________________________________________________
_
batch_normalization_122 (BatchN (None, 120, 160, 48) 192         conv2d_122[0][0]
__________________________________________________________________________________________________
_
activation_122 (Activation)     (None, 120, 160, 48) 0           batch_normalization_122[0][0]
__________________________________________________________________________________________________
_
conv2d_123 (Conv2D)             (None, 120, 160, 12) 5184        activation_122[0][0]
__________________________________________________________________________________________________
_
concatenate_59 (Concatenate)    (None, 120, 160, 108 0           concatenate_58[0][0]
                                                                 conv2d_123[0][0]
__________________________________________________________________________________________________
_
batch_normalization_123 (BatchN (None, 120, 160, 108 432         concatenate_59[0][0]
__________________________________________________________________________________________________
_
activation_123 (Activation)     (None, 120, 160, 108 0           batch_normalization_123[0][0]
__________________________________________________________________________________________________
_
conv2d_124 (Conv2D)             (None, 120, 160, 48) 5184        activation_123[0][0]
__________________________________________________________________________________________________
_
batch_normalization_124 (BatchN (None, 120, 160, 48) 192         conv2d_124[0][0]
__________________________________________________________________________________________________
_
activation_124 (Activation)     (None, 120, 160, 48) 0           batch_normalization_124[0][0]
__________________________________________________________________________________________________
_
conv2d_125 (Conv2D)             (None, 120, 160, 12) 5184        activation_124[0][0]
__________________________________________________________________________________________________
_
concatenate_60 (Concatenate)    (None, 120, 160, 120 0           concatenate_59[0][0]
                                                                 conv2d_125[0][0]
__________________________________________________________________________________________________
_
batch_normalization_125 (BatchN (None, 120, 160, 120 480         concatenate_60[0][0]
__________________________________________________________________________________________________
_
activation_125 (Activation)     (None, 120, 160, 120 0           batch_normalization_125[0][0]
__________________________________________________________________________________________________
_
conv2d_126 (Conv2D)             (None, 120, 160, 60) 7200        activation_125[0][0]
__________________________________________________________________________________________________
_
```

```
average_pooling2d_4 (AveragePoo (None, 60, 80, 60)   0           conv2d_126[0][0]
__________________________________________________________________________________________________
batch_normalization_126 (BatchN (None, 60, 80, 60)   240         average_pooling2d_4[0][0]
__________________________________________________________________________________________________
activation_126 (Activation)     (None, 60, 80, 60)   0           batch_normalization_126[0][0]
__________________________________________________________________________________________________
conv2d_127 (Conv2D)             (None, 60, 80, 48)   2880        activation_126[0][0]
__________________________________________________________________________________________________
batch_normalization_127 (BatchN (None, 60, 80, 48)   192         conv2d_127[0][0]
__________________________________________________________________________________________________
activation_127 (Activation)     (None, 60, 80, 48)   0           batch_normalization_127[0][0]
__________________________________________________________________________________________________
conv2d_128 (Conv2D)             (None, 60, 80, 12)   5184        activation_127[0][0]
__________________________________________________________________________________________________
concatenate_61 (Concatenate)    (None, 60, 80, 72)   0           average_pooling2d_4[0][0]
                                                                 conv2d_128[0][0]
__________________________________________________________________________________________________
batch_normalization_128 (BatchN (None, 60, 80, 72)   288         concatenate_61[0][0]
__________________________________________________________________________________________________
activation_128 (Activation)     (None, 60, 80, 72)   0           batch_normalization_128[0][0]
__________________________________________________________________________________________________
conv2d_129 (Conv2D)             (None, 60, 80, 48)   3456        activation_128[0][0]
__________________________________________________________________________________________________
batch_normalization_129 (BatchN (None, 60, 80, 48)   192         conv2d_129[0][0]
__________________________________________________________________________________________________
activation_129 (Activation)     (None, 60, 80, 48)   0           batch_normalization_129[0][0]
__________________________________________________________________________________________________
conv2d_130 (Conv2D)             (None, 60, 80, 12)   5184        activation_129[0][0]
__________________________________________________________________________________________________
concatenate_62 (Concatenate)    (None, 60, 80, 84)   0           concatenate_61[0][0]
                                                                 conv2d_130[0][0]
__________________________________________________________________________________________________
batch_normalization_130 (BatchN (None, 60, 80, 84)   336         concatenate_62[0][0]
__________________________________________________________________________________________________
activation_130 (Activation)     (None, 60, 80, 84)   0           batch_normalization_130[0][0]
__________________________________________________________________________________________________
conv2d_131 (Conv2D)             (None, 60, 80, 48)   4032        activation_130[0][0]
__________________________________________________________________________________________________
batch_normalization_131 (BatchN (None, 60, 80, 48)   192         conv2d_131[0][0]
__________________________________________________________________________________________________
activation_131 (Activation)     (None, 60, 80, 48)   0           batch_normalization_131[0][0]
__________________________________________________________________________________________________
conv2d_132 (Conv2D)             (None, 60, 80, 12)   5184        activation_131[0][0]
__________________________________________________________________________________________________
concatenate_63 (Concatenate)    (None, 60, 80, 96)   0           concatenate_62[0][0]
                                                                 conv2d_132[0][0]
__________________________________________________________________________________________________
batch_normalization_132 (BatchN (None, 60, 80, 96)   384         concatenate_63[0][0]
__________________________________________________________________________________________________
activation_132 (Activation)     (None, 60, 80, 96)   0           batch_normalization_132[0][0]
__________________________________________________________________________________________________
```

```
conv2d_133 (Conv2D)             (None, 60, 80, 48)   4608        activation_132[0][0]
__________________________________________________________________________________________________
_
batch_normalization_133 (BatchN (None, 60, 80, 48)   192         conv2d_133[0][0]
__________________________________________________________________________________________________
_
activation_133 (Activation)     (None, 60, 80, 48)   0           batch_normalization_133[0][0]
__________________________________________________________________________________________________
_
conv2d_134 (Conv2D)             (None, 60, 80, 12)   5184        activation_133[0][0]
__________________________________________________________________________________________________
_
concatenate_64 (Concatenate)    (None, 60, 80, 108)  0           concatenate_63[0][0]
                                                                 conv2d_134[0][0]
__________________________________________________________________________________________________
_
batch_normalization_134 (BatchN (None, 60, 80, 108)  432         concatenate_64[0][0]
__________________________________________________________________________________________________
_
activation_134 (Activation)     (None, 60, 80, 108)  0           batch_normalization_134[0][0]
__________________________________________________________________________________________________
_
conv2d_135 (Conv2D)             (None, 60, 80, 48)   5184        activation_134[0][0]
__________________________________________________________________________________________________
_
batch_normalization_135 (BatchN (None, 60, 80, 48)   192         conv2d_135[0][0]
__________________________________________________________________________________________________
_
activation_135 (Activation)     (None, 60, 80, 48)   0           batch_normalization_135[0][0]
__________________________________________________________________________________________________
_
conv2d_136 (Conv2D)             (None, 60, 80, 12)   5184        activation_135[0][0]
__________________________________________________________________________________________________
_
concatenate_65 (Concatenate)    (None, 60, 80, 120)  0           concatenate_64[0][0]
                                                                 conv2d_136[0][0]
__________________________________________________________________________________________________
_
batch_normalization_136 (BatchN (None, 60, 80, 120)  480         concatenate_65[0][0]
__________________________________________________________________________________________________
_
activation_136 (Activation)     (None, 60, 80, 120)  0           batch_normalization_136[0][0]
__________________________________________________________________________________________________
_
conv2d_137 (Conv2D)             (None, 60, 80, 48)   5760        activation_136[0][0]
__________________________________________________________________________________________________
_
batch_normalization_137 (BatchN (None, 60, 80, 48)   192         conv2d_137[0][0]
__________________________________________________________________________________________________
_
activation_137 (Activation)     (None, 60, 80, 48)   0           batch_normalization_137[0][0]
__________________________________________________________________________________________________
_
conv2d_138 (Conv2D)             (None, 60, 80, 12)   5184        activation_137[0][0]
__________________________________________________________________________________________________
_
concatenate_66 (Concatenate)    (None, 60, 80, 132)  0           concatenate_65[0][0]
                                                                 conv2d_138[0][0]
__________________________________________________________________________________________________
_
batch_normalization_138 (BatchN (None, 60, 80, 132)  528         concatenate_66[0][0]
__________________________________________________________________________________________________
_
activation_138 (Activation)     (None, 60, 80, 132)  0           batch_normalization_138[0][0]
__________________________________________________________________________________________________
_
global_average_pooling2d_2 (Glo (None, 132)          0           activation_138[0][0]
__________________________________________________________________________________________________
_
dense_2 (Dense)                 (None, 2)            266         global_average_pooling2d_2[0][0]
==================================================================================================
=
Total params: 180,146
Trainable params: 175,058
Non-trainable params: 5,088
```

```
_
None
```
